# Prehabilitation programs for individuals with cancer: a systematic review of randomized-controlled trials

**DOI:** 10.1186/s13643-023-02373-4

**Published:** 2023-11-17

**Authors:** Jose F. Meneses-Echavez, Andrés F. Loaiza-Betancur, Víctor Díaz-López, Andrés M. Echavarría-Rodríguez, Héctor Reynaldo Triana-Reina

**Affiliations:** 1 Division for Health Services, Norwegian Institute of Public Health, Sandakerveien 24C, Building D11, Oslo, Norway; 2 Facultad de Cultura Física, Deporte y Recreación. GICAEDS, Universidad Santo Tomás, Bogotá, Colombia; 3 Universidad de Antioquia. Instituto Universitario de Educación Física, Medellín, Colombia; 4 Grupo de Investigación en Entrenamiento Deportivo y Actividad Física Para La Salud (GIEDAF), Universidad Santo Tomás, Tunja, Colombia

**Keywords:** Cancer, Prehabilitation, Systematic review, Meta-analysis, Exercise

## Abstract

**Background:**

Prehabilitation programs focusing on exercise training as the main component are known as a promising alternative for improving patients’ outcomes before cancer surgery. This systematic review determined the benefits and harms of prehabilitation programs compared with usual care for individuals with cancer.

**Methods:**

We searched CENTRAL, MEDLINE, and EMBASE from inception to June 2022, and hand searched clinical trial registries. We included randomized-controlled trials (RCTs) in adults, survivors of any type of cancer, that compared prehabilitation programs that had exercise training as the major component with usual care or other active interventions. Outcome measures were health-related quality of life (HRQL), muscular strength, postoperative complications, average length of stay (ALOS), handgrip strength, and physical activity levels. Two reviewers independently screened the studies, extracted data, and assessed the risk of bias and the certainty of the evidence.

**Results:**

Twenty-five RCTs (2682 participants) published between 2010 and 2022 met our inclusion criteria. Colorectal and lung cancers were the most common diagnoses. The studies had methodological concerns regarding outcome measurement, selective reporting, and attrition. Five prehabilitation programs were compared to usual care (rehabilitation): combined training, aerobic training, respiratory muscle training plus aerobic training, respiratory muscle training plus resistance training, and pelvic floor training. The studies provided no clear evidence of an effect between groups. We assessed the overall certainty of the evidence as very low, downgraded due to serious study limitations and imprecision.

**Conclusion:**

Prehabilitation programs focusing on exercise training may have an effect on adults with cancer, but the evidence is very uncertain. We have very little confidence in the results and the true effect is likely to be substantially different from these. Further research is needed before we can draw a more certain conclusion.

**Systematic review registration:**

CRD42019125658.

**Supplementary Information:**

The online version contains supplementary material available at 10.1186/s13643-023-02373-4.

## Background

Cancer is a chronic disease in which abnormal cells divide without control, can invade nearby tissues, and can spread to other parts of the body through the blood and lymph systems [1]. GLOBOCAN reported 18.1 million new cases of cancer and 9.6 million deaths in 2018 [2]. Depending on the type of cancer and staging, treatment options include surgery, chemotherapy, radiation therapy, immunotherapy, hematopoietic stem cell transplant, and hormone therapy [1].

Surgery and postoperative care are frequently required during cancer treatment, which results in prolonged inactivity and deconditioning, loss of muscular function, and an increased risk of complications [3]. The lower body is the most affected by this loss of muscle mass, which is larger during the first days of inactivity [4–6]. Exercise training during and after cancer treatment improves quality of life [7], decreases fatigue and depression symptoms [7], and may even lessen tumor activity [8, 9].

Cancer prehabilitation represents “a process on the continuum care that occurs between the time of cancer diagnosis and the beginning of acute treatment. It includes physical and psychological assessments that establish a baseline functional level, identifies impairments, and provides targeted interventions that improve a patient’s health to reduce the incidence and the severity of current and future impairments”[10]. A recent Swedish cohort study showed that fewer postoperative complications and shorter length of stay after abdominal cancer resection were associated with better walking distance, leg strength, grip strength, gait speed, and inspiratory muscle strength [11]. Prehabilitation programs might also improve lean mass, muscular strength, and therefore delay the onset of sarcopenia [12].

Although cancer research has traditionally focused on postoperative exercise (rehabilitation) [4, 13], this might be too late for older individuals, who are at high risk due to their reduced physical capacity, especially before surgery. Individuals with cancer who have adequate muscular strength and cardiorespiratory fitness experience better postsurgical recovery [13]. A recent systematic review conducted by Hamaker and colleagues [14] found small benefits from prehabilitation programs and therefore questioned their relevance. Besides, previous systematic reviews [14, 15] have investigated multimodal prehabilitation programs rather than exercise-focused programs, emphasized the lack of a comprehensive assessment of prehabilitation program reporting, and did not grade the evidence's certainty. These limitations hinder the transferability of research findings into practice.

This systematic review aimed to determine the benefits and harms of prehabilitation programs compared with usual care for individuals with cancer. We assessed the reporting completeness of exercise interventions within the prehabilitation programs along with the certainty of the evidence.

## Methods

This systematic review was conducted following the Cochrane Handbook for Systematic Reviews of Interventions [16] and reported in accordance with the guidelines of the Preferred Reporting Items for Systematic Reviews and Meta-Analyses (PRISMA) statement (Additional file 1) [17]. We registered the protocol in the International Prospective Register of Systematic Reviews (PROSPERO registration number: CRD42019125658).

### Search methods

A research librarian searched Cochrane Central Register of Controlled Trials (CENTRAL), MEDLINE, and EMBASE in November 2019 (from inception—latest update in June 2022). Additional file 2 presents the search strategies. No restrictions were applied for publication date or language. One reviewer (JM) searched for ongoing studies in the WHO ICTRP portal and ClinicalTrials.gov by using free search terms taken from the main search strategies (Additional file 2). Finally, two reviewers (JM and ALB) independently examined the reference lists from relevant publications and key journals and added appropriate titles to the search output.

### Inclusion criteria and study selection

We included randomized-controlled trials (RCTs) in participants older than 13 years old, survivors of any type of cancer, defined according to the Centers for Disease Control and Prevention (CDC), as anyone who has been diagnosed with cancer, from the time of diagnosis through the rest of life [18]. We applied no restrictions regarding nationality, ethnicity, gender, duration of illness, cancer progression status, cancer treatment, or treatment setting. We included prehabilitation programs, in which exercise training was the major component [19]. The information reported by the study authors, along with discussion among the review team, helped us clarify the role of exercise training within the prehabilitation programs. We also accepted for inclusion different training modes, such as aerobic, resistance, and flexibility training, as well as yoga, Qigong, and Tai-Chi [20]. We excluded multimodal interventions (e.g., nutritional therapy or psychological treatments) as well as physiotherapy interventions, such inspiratory muscle training only. RCTs with usual care, sham intervention, or wait-list controls were included.

In order to provide a more comprehensive and clinically relevant set of outcome measures, three reviewers (ALB, VD, and AE) conducted a scoping search of recent systematic reviews and mapped out the outcome measures explored among them. All team members reviewed and discussed the final set of outcomes that were included in this systematic review [19]. We included the following outcomes:

- *Primary outcomes*: Health-related quality of life (HRQoL), muscular strength, and postoperative complications.
- *Secondary outcomes*: average length of stay (ALOS), handgrip strength, physical activity levels (both light and moderate).

We piloted the eligibility criteria in 10% of the anticipated total sample. Once we reached high agreement (> 70%) between pairs of reviewers, we used Rayyan [21] to individually screen citations. Pairs of reviewers screened titles and abstracts, and each relevant full-text article was independently reviewed against the inclusion criteria. We resolved discrepancies through discussion or by involving a third reviewer.

### Data extraction and risk of bias assessment

Pairs of reviewers worked independently to extract data and assess the risk of bias of the included studies with the Cochrane risk of bias version 1 tool [22].

### Completeness of reporting of exercise training interventions in the prehabilitation programs

Two independent reviewers (AE and VD) used the CERT tool (Consensus on Exercise Reporting Template) [23, 24] to assess the reporting completeness of exercise training interventions within the prehabilitation programs. We applied the CERT tool at the intervention level rather than at the study level.

### Data synthesis

One reviewer (ALB) entered the data in Review Manager (RevMan). We calculated risk ratio (RR) and its 95% confidence interval (CI) for binary outcomes, whereas continuous data were expressed as group post-test means and standard deviations (SDs). We presented effect sizes preferentially as mean difference (MD) and 95% CIs, but when different scales were used to measure the same outcome, we calculated standardized mean difference (SMD). If needed, we used the quantile estimation (QE) method to estimate the sample mean and standard deviation from median, minimum, and maximum values, and sample size [25, 26].

We used DerSimonian-Laird random effects models because of the heterogeneity across studies [15]. We used the *I*^*2*^ statistic to quantify the proportion of variability attributable to between-study heterogeneity [16]. Besides, we registered all relevant data reported in the studies, and organized them as clinically relevant follow-up periods of before surgery, after surgery or post-intervention, four weeks, eight to nine weeks, 12 weeks, and 24 to 26 weeks post-intervention.

### Certainty of the evidence

We followed the GRADE (Grading of Recommendations, Assessment, Development and Evaluations) approach to assess the certainty (or quality) of evidence in six major outcomes [27]. The GRADE approach considers the risk of bias and the body of literature to rate certainty into one of four levels:

- *High*: We are very confident that the true effect lies close to that of the estimate of the effect.
- *Moderate*: We are moderately confident in the effect estimate: The true effect is likely to be close to the estimate of the effect, but there is a possibility that it is substantially different.
- *Low*: Our confidence in the effect estimate is limited: The true effect may be substantially different from the estimate of the effect.
- *Very low*: We have very little confidence in the effect estimate: The true effect is likely to be substantially different from the estimate of effect

## Results

### Search results

**Fig. 1:**
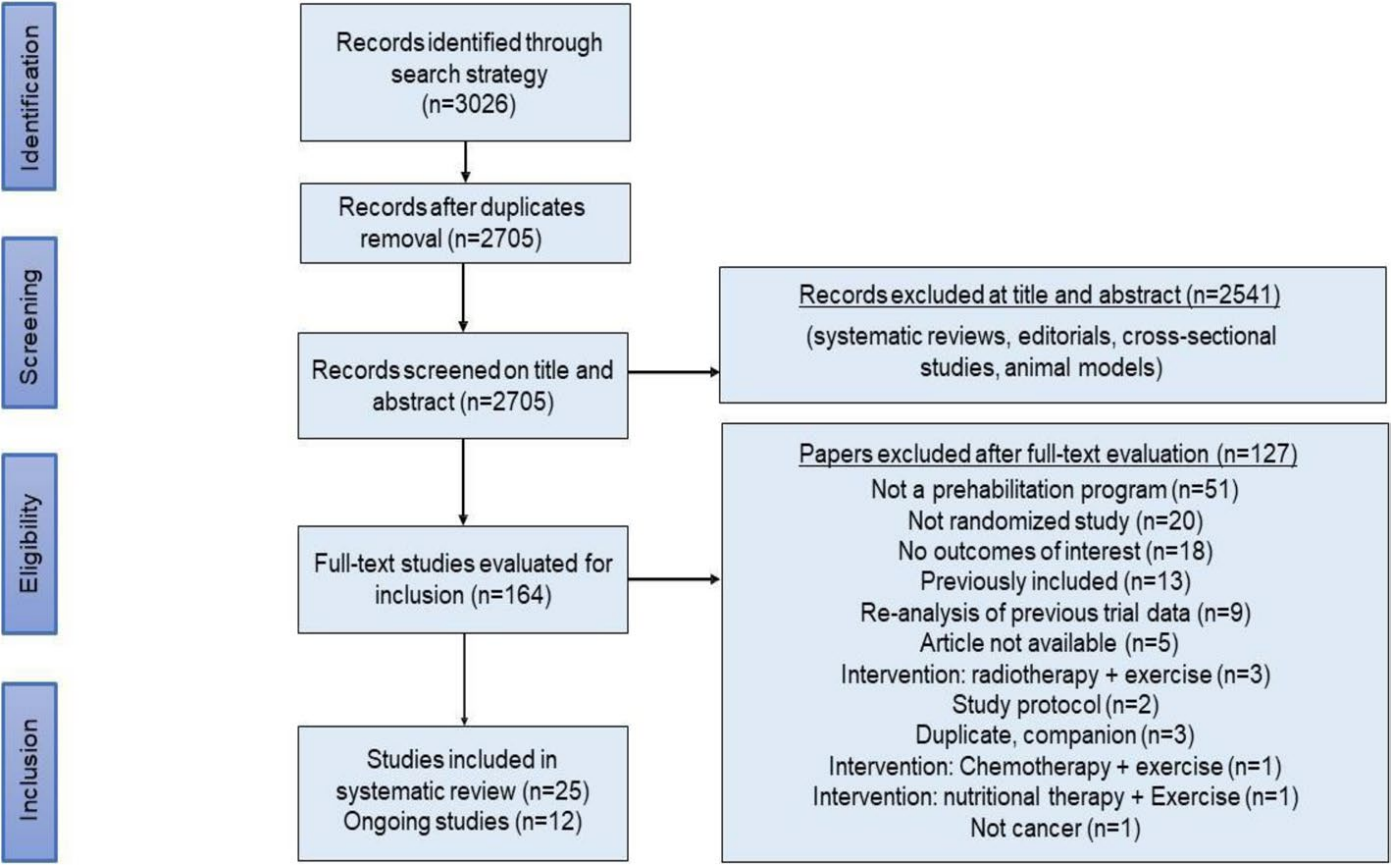
Study flow diagram for the screening process

The searches yielded 3026 records. After 321 duplicates were removed, 2705 records remained to be screened. We excluded 2541 records on title and abstract screening. We assessed 164 full‐text articles for eligibility and excluded 127 full‐text articles. Twenty-five original studies met our inclusion criteria (Fig. 1). Additional file 3 presents the list of excluded studies, and Additional file 4 describes the twelve ongoing studies.

### Characteristics of the included studies

All studies used a two-arm parallel design except for Laurienzo 2013 [28]. The studies included 2682 participants (mean age 65 years). The publication year ranged from 2010 to 2022. Colorectal cancer was the most common diagnosis (11 studies, 44%) [13, 29–38], followed by lung (five studies, 20%) [39–43] and prostate cancer (four studies, 16%) [28, 44–46]. Most studies compared prehabilitation programs with usual care (17 studies, 68%) [28, 29, 33, 35–43, 46–50], while three studies (21%) [30, 32, 34] compared prehabilitation with rehabilitation (i.e., the combination of aerobic and resistance training after surgery). Other studies used controls groups of pelvic floor training [44, 45] walking training [31], no intervention [51], or inspiratory muscle training [13] (Table 1).

### Description of the interventions

The studies evaluated heterogeneous prehabilitation programs, with important differences in terms of exercise modalities and prescription rules. Eight studies (32%) evaluated the effects of combined training of moderate-intensity continuous training (MICT) and resistance training [30–32, 34, 40, 43, 45, 48], while three studies (12%) evaluated the combination of HIIT and resistance training [29, 35, 39]. Two studies (8%) intervened with high-intensity interval training (HIIT) [33, 47] and MICT [37, 49], respectively. Three studies (12%) combined MICT and respiratory muscle training [38, 41, 42] or combined training and respiratory muscle training [11, 13, 51]. One study [50] intervened with resistance training and respiratory muscle training and the remaining three studies (12%) evaluated pelvic floor training [28, 44, 46].

Overall, the prehabilitation programs comprised an initial warm-up period of 5 to 10 min, followed by 30 min of combined training (i.e., aerobic and resistance training), aerobic training alone, or pelvic floor exercises, followed by a cool-down period of 5–10 min. The prehabilitation programs lasted four weeks on average (SD 2.9 weeks, ranging from one to 14 weeks), with each session lasting 49 min (SD 16 min). The average number of sessions per week was 3.5 (SD 1.3). Prehabilitation was supervised in eleven studies (44%) [13, 33, 35, 36, 39, 41–43, 46, 47, 51], and facilitated by either therapists (32%) [13, 35, 36, 39, 42, 48, 50, 51] or by mixed groups of healthcare providers. Eight studies (32%) were conducted in mixed settings (home/clinic) [13, 29, 30, 32, 35, 43, 48, 50] and at clinics or hospitals [33, 39, 41, 42, 44, 46, 47, 51].

The reporting of training intensity varied markedly. Three studies [36, 38, 48] reported moderate to high intensity in the Borg scale. The intensity of aerobic training ranged between 40 and 85% of the maximum heart rate [13, 30, 33, 34, 40, 44, 45, 47]. Other studies reported intensities of 80% and 100% peak workload [39, 43] or other methods, while eight studies did not report intensity data [28, 37, 41, 42, 44, 46, 50, 51]. Four studies [28, 44–46] evaluated pelvic floor training performed at moderate to high intensity (two to four times a week), 30–60 min/session, accompanied in most cases by electrostimulation. Yet the studies reported incomplete information about the implementation of these interventions.

Adherence varied between 75 and 100% in nine studies [13, 29, 30, 33, 34, 36, 39, 48, 50] and from 50 to 75% in five studies [32, 37, 42, 43, 45]. Carli 2010 [30] reported an adherence of 16%, while the remaining ten studies did not provide data on adherence. Additional file 5 provides further details on the prehabilitation programs.

### Completeness of reporting of the exercise training interventions in the prehabilitation programs

**Fig. 2:**
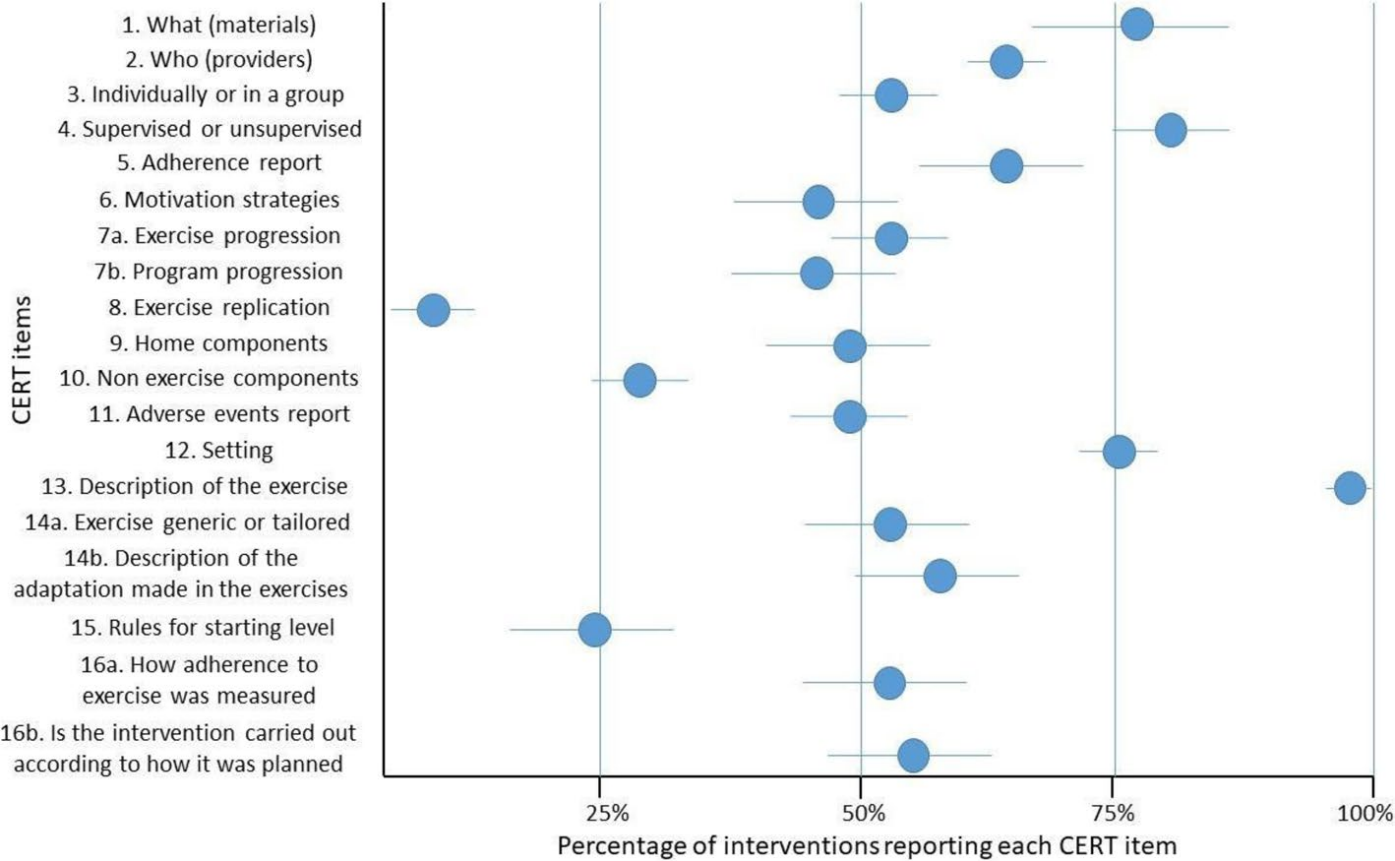
Completeness of reporting of the exercise training interventions: total sample of 25 exercise training interventions. Final version of the CERT checklist used in this study (16 items): 1. What (materials), 2. Who (provider), 3. Individually or in a group, 4. Supervised or unsupervised, 5. Adherence report, 6. Motivation strategies, 7. a. Exercise progression, 7. b. Program progression, 8. Exercise replication, 9. Home components, 10. Non exercise components, 11. Adverse events report, 12. Setting, 13. Description of the exercise, 14. a. Exercises generic or tailored?, 14. b. Description of the adaptation made in the exercises, 15. Rules for starting level, 16. a. How adherence to exercise was measured, 16. b. Is the intervention carried out according to how it was planned?

The included studies reported 25 exercise training interventions. Completeness of reporting ranged from 8 to 96% across the CERT items (Fig. 2). At least five interventions reported ≥ 15 out of the 19 CERT items (about 80% of total reporting). The most reported items were type of exercise equipment (item 1, 80%), supervision (item 4, 84%), setting (item 12, 76%), and the detailed description of the exercise intervention (item 13, 96%). In contrast, the least reported items were the description of each exercise to enable replication (item 8, 8%), the decision rules for determining the starting level (item 15, 24%), and the description of any non-exercise components (item 10, 32%). The reporting of the remaining items varied between 44 and 68%.

Exercise interventions in participants with colorectal cancer had the highest level of reporting relative to those in lung cancers. Combined training was the exercise mode with the most complete reporting. Additional file 6 presents the subgroup analysis undertaken for the completeness of reporting.

### Outcome measures

#### Primary outcomes

##### Health-related quality of life (HRQoL)

Ten studies (40%) [13, 28, 32–35, 37, 42–46, 48, 50] measured this outcome. The tools most used were the Short Form Health Survey (SF-36) and the European Organization for Research and Treatment of Cancer quality of life questionnaire (EORTC QLQ-C30), used in five [28, 32–34, 48] and three studies [13, 37, 42], respectively. The minimal clinically important difference (MCID) for SF-36 was five points for both physical and mental component summary scores [52]. The MCID of the EORTC QLQ-C30 for between-group change over time ranged from 4 to 11 points (improvement) and from 18 to 4 points (deterioration) across all scales [53].

##### Muscular strength

Five studies (20%) measured muscular strength [13, 37, 43, 45, 48] using different tests, such as the elbow flexion and extension test [45], the "Senior Fitness Test" [43], and the chair rise test [13]. The MCID of the Chair rise test was five repetitions [54]. We could not identify MCIDs for the remaining tests (Table 1).

**Table 1:** Characteristics of the included studies (*n* = 25)

Study ID, year Country (Trial registry record)	Participants Type of cancer (N, age)	Intervention and control	Outcomes
Banerjee 2018 [47] UK (JSCC-D-17–00896)	Bladder N: 60 Int: 30, age (yr): mean 71 (SD 6) Con: 30, age (yr): mean 72 (SD 8)	Int: Aerobic training (HIIT) Con: Usual care	Postoperative complications ALOS
Berkel 2022 [29] Netherlands (NTR4032)	Colorectal N: 57 Int: 28, age (yr): mean 74 (SD 7) Con: 29, age (yr): mean 73 (SD 6)	Int: Combined training (HIIT/resistance) Con: Usual care	Postoperative complications ALOS
Bousquet-Dion 2018 [30] Canada (NCT02586701)	Colorectal N: 63 Int: 37, age (yr): median 74 (IQR 67 to 78) Con: 26, age (yr): median 71 (IQR 54 to 74)	Int: Combined training (MICT/Resistance) Con: Rehabilitation	Postoperative complications Handgrip strength Physical activity levels
Carli 2010 [31] Canada (NCT00227526)	Colorectal N: 112 Int: 58, age (yr) mean 61 (SD 16) Con: 54, age (yr) mean 60 (SD 15)	Int: Combined training (MICT/Resistance) Con: Rehabilitation (walk/breathing group)	Postoperative complications ALOS Physical activity levels
Carli 2020 [32] Canada (NCT02502760)	Colorectal N: 110 Int: 55, age (yr): median 78 (IQR 72 to 82) Con: 55, age (yr): median 82 (IQR 75 to 84)	Int: Combined training (MICT/Resistance) Con: Rehabilitation	HRQoL Postoperative complications ALOS Physical activity levels
Centemero 2010 [44] Italy (NR)	Prostate N: 118 Int: 59, age (yr): 60 (range: 48–68) Con: 59, age (yr): 57 (range: 46–67)	Int: Pelvic floor training Con: Pelvic floor training (rehabilitation5)	HRQoL
Dronkers 2010 [13] Netherlands (NR)	Colon N: 42 Int: 22, age (yr): mean 71 (SD 6) female 18% Con: 20, age (yr): mean 68 (SD 6), female 35%	Int: Combined training (MICT/Resistance) and respiratory muscle training Con: Rehabilitation (home-based exercise advice plus inspiratory muscle training advice)	HRQoL Muscular strength Postoperative complication Physical activity levels
Dunne 2016 [33] United Kingdom (NCT01523353)	Colorectal N: 34 Int: 19, age (yr): median 61 (IQR 56 to 66) Con: 15, age (yr): median 62 (IQR 53 to 72)	Int: Aerobic training (HIIT) Con: Usual care	HRQoL Postoperative complications
Gillis 2014 [34] Canada (NCT01356264)	Colorectal N: 77 Int: 38, age (yr): mean 66 (SD 14) Con: 39, age (yr): mean 66 (SD 9)	Int: Combined training (MICT/Resistance) Con: Rehabilitation	HRQoL Postoperative complications ALOS Physical activity levels
Gloor 2022 [35] Switzerland (NCT02746731)	Colorectal N: 107 Int: 54, age (yr): median 66 (24–90); female 56% Con: 53, age (yr): median 65 (29–86); female 40%	Int: Combined training (HIIT, resistance) Con: Usual care	HRQoL Postoperative complications ALOS Handgrip strength Physical activity levels
Heiman 2021 [49] Sweden (NCT02560662)	Breast N: 316Int: 148, age (yr): 61 (52–68); female all Con: 168, age (yr): median 63 (54–71)	Int: Aerobic training (MICT) Con: Usual care	Postoperative complications ALOS Physical activity
Karlsson 2019 [11, 49] Sweden (NCT02895464)	Colorectal N: 21 Int: 10, age (yr): median 83 (76–85); female 60% Con: 11, age (yr): median 74 (73–76); female 64%	Int: Combined training (HIIT/resistance) and inspiratory muscle training Con: Usual care	Postoperative complications ALOS
Lai 2017 [42] China (ChiCTR-IOR-16008109)	Lung N: 101 Int: 51, age (yr): 64 Con: 50, age (yr): 65; female 23%	Int: Aerobic training (MICT) and respiratory muscles training Con: Usual care	HRQoL Postoperative complications
Laurienzo 2013 [28, 42] Brasil (NR)	Prostate N: 49 Int: 17, age (yr): mean 62 (SD 8) Con 1: 17, age (yr): mean 64 (SD 7) Con 2: 15, age (yr): mean 60 (SD 8)	Int: Pelvic floor training Con 1: Pelvic floor training plus electrical stimulation Con 2: Usual care	HRQoL
Licker 2016 [39] Switzerland (NCT01258478)	Lung N: 151 Int: 74, age (yr): 64 Con: 77, age (yr): 64	Int: Combined training (HIIT/ resistance) Con: Usual care	Postoperative complications ALOS
Liu 2019 [40] China (NCT03068507)	Lung N:73 Int: 37, age (yr): mean 56 (SD 10) Con: 36, age (yr): mean 56 (SD 9)	Int: Combined training (MICT/ resistance) Con: Usual care	Postoperative complications ALOS
Moug 2019 [37] Scotland (ISRCTN 62859294)	Colorectal N: 40 Int: 18, age (yr): mean 65 (SD 11) female 25% Con: 22, age (yr): mean 66 (SD 9)	Int: Aerobic training (MICT) Con: Usual care	HRQoL Muscular strength Postoperative complications ALOS Physical activity levels
Ocampo-Trujillo 2014 [46] Colombia (NR)	Prostate N: 16 Int: 8, age (yr): 57 Con: 8, age (yr): 66	Int: Pelvic floor training Con: Usual care	HRQoL
Onerup 2022 [38] Sweden (NCT02299596)	Colorectal N: 668 Int: 317, age (yr): mean 69 (SD 11) Con: 351, age (yr): mean 68 (SD 11)	Int: Aerobic training (MICT) inspiratory muscle training (IMT) Con: Usual care	Postoperative complications ALOS
Pehlivan 2011 [41] Turkey (NR)	Lung N: 60 Int: 30, age (yr): 54 Con: 30, age (yr): 55	Int: Respiratory muscles training and aerobic training (MICT) Con: Usual care	Postoperative complications ALOS
Peng 2021 [50] China (ChiCTR-ONRC-14005096)	Colorectal N: 213 Int: 109, age (yr): mean 63 (SD 2) Con: 104, age (yr): mean 62 (SD 3)	Int: Resistance training and respiratory muscle training Con: Usual care	HRQoL Postoperative complications ALOS Handgrip strength
Santa Mina 2018 [45] Canada (NCT02036684)	Prostate N:61 Int: 33, age (yr): mean 61 (SD 8) Con: 28, age (yr): mean 62 (SD 7)	Int: Combined training (MICT/ resistance) Con: Pelvic floor training	HRQoL Muscular strength Postoperative complications ALOS Handgrip strength Physical activity levels
Sebio-García 2017 [43, 45] Spain (NCT01963923)	Lung N:19 Int: 9, age (yr): mean 71 (SD 6) Con: 10, age (yr): mean 69 (SD 9)	Int: Combined training (MICT/resistance) Con: Usual care	HRQoL Muscular strength Postoperative complications ALOS
Steffens 2021 [48] Australia (ACTRN12617001129370)	Abdominal cancer N: 22 Int: 11, Age 62 (yr) (range 48 to 72) Con: 11, age (yr) 66 (range 46 to 70)	Int: Combined training (MICT/resistance) Con: Usual care	HRQoL Muscular strength Postoperative complications ALOS Physical activity
Yamana 2015 [51] Japan (UMIN No. 00006216)	Esophageal N: 60 Int: 30, age (yr) 68 (SD 7 64) Con: 30, age (yr): 65 (SD 9)	Int: Combined training (MICT/Resistance) and respiratory rehabilitation Con: No intervention	Postoperative complications

*ALOS* Average length of stay, *Con* Control, *HIIT* High-intensity interval training, *HRQoL* Health-related quality of life, *Int* Intervention, *IQR* Interquartile range, *MICT* Moderate-intensity continuous training, *N* Number of participants analyzed in the study, *NR* Not reported, *PR* Preoperative respiratory rehabilitation, *SD* Standard deviation, *UK* United Kingdom, *yr* Year

##### Postoperative complications

This was the most reported outcome (22 studies, 88%) [13, 29–43, 45, 47–51]. Most of the studies used The Clavien-Dindo Classification (14 studies, 56%).

#### Secondary outcomes

##### Average length of stay (ALOS)

Eighteen studies (72%) [13, 29, 31, 32, 34–41, 43, 45, 47–50] reported this outcome using administrative data. Three days has been proposed as a valid MCID [55].

##### Handgrip strength

Santa Mina 2018 [45] measured this outcome using dynamometry (Sammons Preston, Bolingbrook, IL, USA), while Peng 2021 [49] used a hydraulic dynamometer (Saehan Corporation, Masan, Korea). The MCID varied between 5 to 6.5 kg [56, 57].

##### Physical activity levels

Nine studies (33%) measured this outcome. The Community Health Activities Model Program for Seniors (CHAMPS) physical activity questionnaire was the most used tool (five studies, 20%) [30–32, 34, 45]. The MCID (min/day) of the CHAMPS questionnaire was 30 for light activities, 28 for moderate exercise, and 39 for light to moderate activities [58]. When expressed as kcal/day, the MCID values were as follows: 109 for light activity, 236 for moderate activities, and 231 for light to moderate activities [58]. The MCID of IPAQ (International Physical Activity Questionnaire) was 13 to 58 min/wk [59].

Table 2 describes the follow-up periods reported for each outcome in the included studies.

**Table 2:** Outcome measures in the included studies: measurement tools and follow-up periods

Outcome	Measurement tools	Follow-up periods: number of studies
Health-related quality of life	SF-36 [28, 32–34, 48] SF-36 v2 [43] ICS male SF [44] FACT-P [45] EORTC QLQ-C30 [42] SF-12 v2 [46] QOR-40 [50]	Before surgery: seven studies [13, 32, 34, 37, 43, 45, 48] Post-surgery: two studies [43, 48] 4 weeks post-intervention: seven [28, 32–34, 42, 44, 45] 8 weeks post-intervention: two studies [34, 46] 12 weeks post-intervention: four studies [28, 43–45] 24 weeks post-intervention: one study [28] 26 weeks post-intervention: one study [45]
Muscular strength	Chair rise test [13] Elbow flexion and extension [45] Senior Fitness Test [43, 45] Sit-to-stand test [37, 48] Quadriceps strength test [48]	Before surgery: five studies [13, 43, 45] Post-surgery: two studies [43, 48] 4 weeks post-intervention: one study [45] 9 weeks post-intervention: one study [45] 12 weeks post-intervention: one study [43] 26 weeks post-intervention: one study [45]
Postoperative complications	The Clavien-Dindo Classification [28, 34, 35, 37, 38, 40] [30, 31, 33, 34, 40, 45] Melbourne Group Scale [43] ICC [32] TMM [31] Admin data [13]	1-week post-intervention: six studies [13] 4 weeks post-intervention: eleven studies [30, 32, 39, 40, 42] 8 weeks post-intervention: one study [34] 9 weeks post-intervention: one study [33] 12 weeks post-intervention: four studies [33, 43] 26 weeks post-intervention: one study [45]
Average length of stay	Administrative data	1-week post-intervention: five studies [32, 38–40, 47] 4 weeks post-intervention: eight studies [29, 32, 38–40, 47, 49, 50] 9 weeks post-intervention: one study [31] 12 weeks post-intervention: two studies [38, 43] 26 weeks post-intervention: one study [45]
Handgrip strength	Handgrip dynamometry [45] OJO: agregar el test de Peng [50] hydraulic dynamometer	Before surgery: two studies [45, 50] Post-surgery: one stuy [50] 4 weeks post-intervention 12 weeks post-intervention 26 weeks post-intervention
Physical activity levels	CHAMPS [30–32, 34, 45] LASA Physical Activity Questionnaire [13] Accelerometer [37, 49] SGPALS [49] IPAQ [48]	Before surgery: eight studies [13, 30–32, 34, 37, 45, 48] Post-intervention: one study [48] 4 weeks post-intervention: six studies [30–32, 34, 45, 49] 8 weeks post-intervention: two studies [42, 43] 9 weeks post-intervention: one study [31] 26 weeks post-intervention: one study [45, 48]

*CHAMPS* The Community Healthy Activities Model Program for Seniors, *EORTC QLQ-C30* The European Organization for Research and Treatment of Cancer Quality of Life Questionnaire Core 30, *FACT-P* The Functional Assessment of Cancer Therapy-Prostate, *ICC* The Comprehensive Complication Index, *ICS SF* the International Continence Society male Short Form, *IPAQ* International Physical Activity Questionnaire, SF-12 / *SF-36* The Short FormHealth Survey, *SF-36 v2* (version 2), *SGPALS* The Saltin–Grimby Physical Activity Level Scale, *The Clavien-Dindo* Classification for surgical complications, *TMM* Thoracic morbidity and mortality

In order to provide a comprehensive overview of the body of evidence, we mapped out additional outcome measures reported in the included studies that were not considered in the current systematic review (Additional file 7).

#### Allocation

Twenty-three studies (92%) described adequate methods of random sequence generation [13, 29–44, 46–50, 60], while the remaining two studies (8%) were rated as unclear risk of selection bias given no further information [45, 51]. Five studies (20%) lacked information about allocation concealment and were assessed as being at unclear risk of bias for this domain [13, 28, 36, 41, 46]. Twenty studies (80%) described allocation concealment clearly, so these studies were judged at low risk of bias for this domain [29, 30, 32–35, 37–40, 42–45, 47, 48, 50, 51, 60].

#### Blinding

*Objective outcomes*: We rated blinding of participants, personnel, and outcome assessment as low risk in the twenty-two studies (88%) that reported objective outcomes [13, 29–43, 45, 47–51, 60].

##### Objective outcomes

We rated blinding of participants, personnel, and outcome assessment as low risk in the twenty-two studies (88%) that reported objective outcomes [13, 29–43, 45, 47–51, 60].

##### Subjective outcomes

All but eight studies reported subjective outcomes [29, 36, 38–41, 47, 51]. Blinding of participants and personnel was not possible due to the nature of prehabilitation, so the studies were judged to be at high risk of bias. Six studies (24%) were at high risk of detection bias, as these included self-reported outcomes [28, 31, 35, 45, 46, 49] while the remaining eleven studies (44%) described adequate blinding of outcome assessment [13, 30, 32–34, 37, 42–44, 48, 50].

#### Incomplete outcome data

We judged eighteen studies (72%) to be at low risk of attrition bias (low overall attrition) [13, 28, 29, 32–36, 38, 39, 41, 42, 44, 46–50]. Five studies (20%) had high risk of bias (uneven attrition across groups) [30, 31, 43, 45, 51], whereas Moug 2019 [37] and Liu 2019 [40] were rated as unclear risk due to incomplete information on the rates and reasons for participants being excluded from the analysis in each group.

#### Selective reporting

Four studies (16%) [28, 35, 42, 51] had high risk of bias due to either not reporting all outcomes stated in their protocol or missing data. Eighteen studies (72%) reported all pre-specified outcomes and were judged at low risk of reporting bias [13, 30–34, 36–39, 41, 43–48, 50, 60], while the remaining three studies (12%) were rated as unclear risk of bias [29, 40, 49].

#### Other bias

**Fig. 3:**
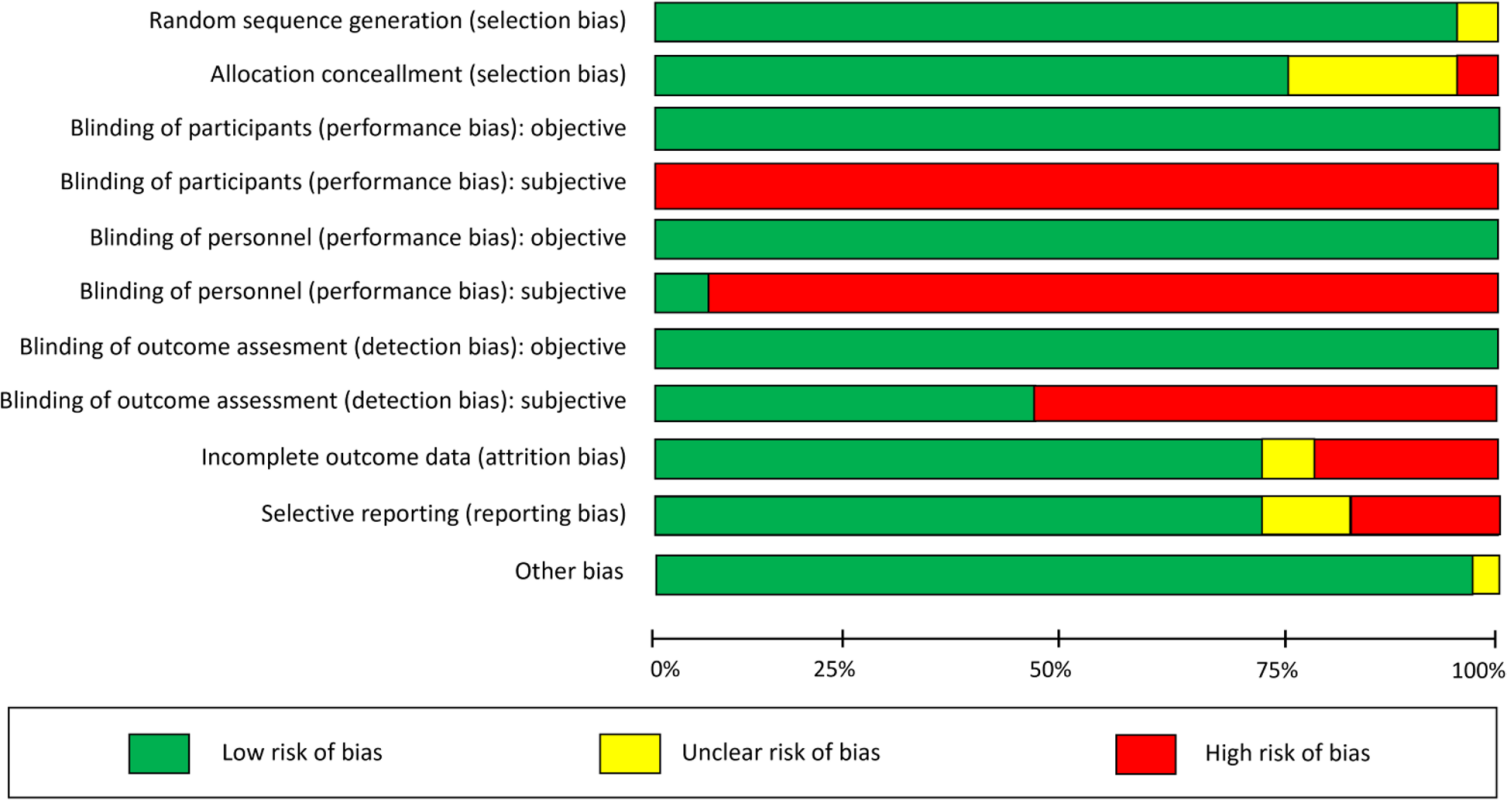
Risk of bias graph: review authors' judgements about each risk of bias item presented as percentages across all included studies

Twenty-four studies (96%) were at low risk of other bias [28–50, 60], only Yamana 2015 [51] was rated as unclear risk due to lack of baseline comparability (Fig. 3).

### Effects of prehabilitation programs

We present the effect of prehabilitation programs per comparison (combined training vs rehabilitation/usual care, resistance training plus respiratory muscle training vs usual care, HIIT vs usual care, respiratory muscle training plus aerobic training vs usual care, pelvic floor training vs usual care), sorted by primary and secondary outcomes.

In most cases, a meta-analysis was deemed inappropriate due to the different follow-up periods, the use of measurement tools that impeded any standardization of the effect estimates, and considerable clinical heterogeneity. We underline that readers should interpret effect estimates with caution given the evidence's very low certainty (Tables 3, 4 and 5).

**Table 3:** Summary of findings table for the comparison: combined training vs rehabilitation/usual care

Prehabilitation programs for individuals with cancer					
**Population:** adults with lung, esophageal, prostate, and colorectal cancer **Intervention:** combined training **Comparison:** rehabilitation, usual care **Setting:** Mixed (home and clinic / hospital)					
Outcomes	Relative effect (95% CI)	**Anticipated absolute effect**^**a**^ **(95% CI)**		Nº of participants (studies)	Certainty of the evidence (GRADE)
		Assumed risk with control	Assumed risk with intervention		
**HRQoL (physical component)**^**b**^ before surgery	MD -2.46 (-7.88 to 2.95)	49 to 59 points	Mean HRQoL in intervention was 2.46 lower (7.9 lower to 2.9 higher)	174 (3 RCTs)	⨁◯◯◯ VERY LOW ^h,i^
**HRQoL (mental component)**^**b**^ before surgery	MD -2.82 (-8.60 to 2.95)	48 to 70 points	Mean HRQoL in intervention was 2.82 lower (8.6 lower to 2.9 higher)	174 (3 RCTs)	⨁◯◯◯ VERY LOW ^h,i^
**HRQoL (overall score)**^**b**^ before surgery	Not estimable	-	-	61 (1 RCT)	⨁◯◯◯ VERY LOW ^j,k^
**HRQoL (overall score)**^**b**^ long term (up to 26 weeks)	Not estimable	-	-	157 (3 RCTs)	⨁◯◯◯ VERY LOW ^j,l^
**Muscular strength:** (before surgery up to 26 weeks)^c^	Not estimable	-	-	162 (4 RCTs)	⨁◯◯◯ VERY LOW ^h,i^
**Postoperative complications: Grade 1** (4 weeks)^d^	RR 1.06 (0.88 to 1.29)	6 per 100	6 per 100 (5 fewer to 8 more)	300 (4 RCTs)	⨁◯◯◯ VERY LOW ^j,k^
**Postoperative complications: Grade 2** (4 weeks)^d^	RR 0.76 (0.48 to 1.18)	1 per 10	1 per 10 (1 fewer to 2 more)	300 (4 RCTs)	⨁◯◯◯ VERY LOW ^j,k^
**Postoperative complications: Grade 3** (4 weeks)^d^	RR 2.78 (0.76 to 10.23)	1 per 10	3 per 10 (1 fewer to 5 more)	300 (4 RCTs)	⨁◯◯◯ VERY LOW ^j,k^
**Postoperative complications: Grade 4** (4 weeks)^d^	RR 1.02 (0.27 to 3.85)	1 per 10	1 per 10 (1 fewer to 4 more)	164 (2 RCTs)	⨁◯◯◯ VERY LOW ^j,k^
**Average length of stay** (4 weeks)^e^	MD -0.01 (-0.56 to 0.54)	Mean stay ranged in control groups from 5 to 10 days	Mean stay in the intervention group was 0.01 shorter (0.56 shorter to 0.54 longer)	391 (4 RCTs)	⨁◯◯◯ VERY LOW ^j,l^
**Handgrip strength** long term (up to 26 weeks) ^f^	Not estimable	-	-	274 (2 RCTs)	⨁◯◯◯ VERY LOW ^j,k^
**Physical activity levels: light** (before surgery)^g^	MD 2.59 (-9.7 to 14.8)	Mean levels ranged in control groups from 15 to 20	Mean levels in the intervention groups was 2.6 higher (10 lower to 15 higher)	150 (2 RCTs)	⨁◯◯◯ VERY LOW ^j,k^
**Physical activity levels: light** (4 weeks)^g^	MD -1.08 (-7.23 to 5.07)	Mean levels ranged in control groups from 12 to 18	Mean levels in the intervention groups was 1 lower (7 lower to 5 higher)	131 (2 RCTs)	⨁◯◯◯ VERY LOW ^j,k^
**Physical activity levels: moderate** (before surgery) ^g^	MD 14.45 (12.8 to 16.1)	Mean levels ranged in control groups from 5 to 14	Mean levels in the intervention groups was 14 higher (13 higher to 16 higher)	150 (2 RCTs)	⨁◯◯◯ VERY LOW ^j,k^
**Physical activity levels: moderate** (4 weeks) ^g^	MD 0.15 (-3.03 to 3.33)	Mean levels ranged in control groups from 12 to 16	Mean levels in the intervention groups was 0.15 higher (3 lower to 3 higher)	131 (2 RCTs)	⨁◯◯◯ VERY LOW ^i,j^

^a^The risk in the intervention group (and its 95% confidence interval) is based on the assumed risk in the comparison group and the relative effect of the intervention (and its 95% CI). *CI* Confidence interval, *HRQoL* Health-related quality of life, *MD* Mean Difference, *RR* Risk ratio

^b^International Continence Society (ICS) ICS male questionnaire; Short Form Health Survey (SF-36 and -12); The European Organization for Research and Treatment of Cancer Quality of Life Questionnaire Core 30 (EORTC QLQ-C30): higher scores indicate better functioning (scaled from 0 to 100); The Functional Assessment of Cancer Therapy-Prostate (FACT-P): high scores indicate worse physical function

^c^Elbow flexion and extension strength: higher mobilized weight indicates higher levels of strength

^d^The Clavien-Dindo Classification; The Comprehensive Complication Index (ICC): high grades indicate worse outcome

^e^Administrative data: high number of days indicate worse response to intervention

^f^Handgrip dynamometry: higher levels indicate better outcome

^g^The Community Healthy Activities Model Program for Seniors (CHAMPS): high scores indicate higher levels of physical activity (light, moderate, and vigorous)

^h^Downgraded by two levels due to no blinding of personnel and outcome measurement (detection bias), and attrition bias

^i^Downgraded by one level due to small sample size and wide confidence intervals (imprecision)

^j^Downgraded by two levels due to no blinding of personnel (performance bias), and selective outcome reporting (reporting bias)

^k^Downgraded by one level due to small sample size (imprecision)

^l^Downgraded by one level due to wide confidence intervals (imprecision)

**Table 4:** Summary of findings table for the comparison: aerobic training (HIIT or MICT) vs usual care

Prehabilitation programs for individuals with cancer					
**Population:** adults with colorectal, bladder, and breast cancer **Intervention:** aerobic training (HIIT or MCIT) **Comparison:** usual care **Setting:** clinic/hospital					
Outcomes	Relative effect (95% CI)	**Anticipated absolute effect**^**a**^ **(95% CI)**		Nº of participants (studies)	Certainty of the evidence (GRADE)
		Assumed risk with control	Assumed risk with intervention		
**HRQoL **^**b**^ (4 weeks)	SF-36, MD 13 (-5 to 30)	59 points	Mean HRQoL in intervention was 13 higher (5 lower to 30 higher)	34 (1 RCT)	⨁◯◯◯ VERY LOW ^e,f^
**HRQoL **^**c**^ (12 weeks)	FACT-C, MD 0.9 (-6.2 to 8.0)	62 points	Mean HRQoL in intervention was 0.9 higher (6 lower to 8 higher)	48 (1 RCT)	⨁◯◯◯ VERY LOW ^e,f^
**Muscular strength** (12 weeks): sit-to-stand test, no. completed in 30 secs	MD -0.6 repetitions (-3.3 to 2.2)	11 repetitions	Mean no. repetitions in intervention was 0.6 lower (3 lower to 2 higher)	48 (1 RCT)	⨁◯◯◯ VERY LOW ^e,f^
**Postoperative complications: All grades **^**d**^ (4 to 12 weeks)	Not estimable	-	Evidence of no difference between groups	513 (4 RCTs)	⨁◯◯◯ VERY LOW ^g,h^
**Average length of stay** (1 to 4 weeks)	Not estimable	-	Evidence of no difference between groups	537 (4 RCTs)	⨁◯◯◯ VERY LOW ^g,h^
**Physical activity levels** (4 weeks)	Not estimable	-	Evidence of no difference between groups	448 (2 RCTs)	⨁◯◯◯ VERY LOW ^g,h^

^a^The risk in the intervention group (and its 95% confidence interval) is based on the assumed risk in the comparison group and the relative effect of the intervention (and its 95% CI). *CI* Confidence interval, *HRQoL* Health-related quality of life, *MD* Mean Difference, *RR* Risk ratio

^b^Short Form Health Survey (SF-36): higher scores indicate worse physical function

^c^Functional Assessment of Cancer Therapy – Colorectal (FACT-C): higher scores mean better quality of life

^d^The Clavien-Dindo Classification: higher grades indicate worse outcome

^e^Downgraded by one level due to incomplete outcome data (attrition bias)

^f^Downgraded by two levels due to small sample size and wide confidence intervals (imprecision)

^g^Downgraded by two levels due to selection bias, selective outcome reporting, and incomplete outcome data (attrition bias)

^h^Downgraded by one level due to wide confidence intervals (imprecision)

**Table 5:** Summary of findings table for the comparison: respiratory muscle training + aerobic training vs usual care

Prehabilitation programs for individuals with cancer					
**Population:** adults with lung and colorectal cancer **Intervention:** respiratory muscle training plus aerobic training **Comparison:** usual care **Setting:** clinic/hospital					
Outcomes	Relative effect (95% CI)	**Anticipated absolute effect**^**a**^ **(95% CI)**		Nº of participants (studies)	Certainty of the evidence (GRADE)
		Assumed risk with control	Assumed risk with intervention		
**HRQoL (overall) **^**b**^ (4 weeks)	MD 1.1 (-1.9 to 4.2)	69 points	Mean HRQoL in intervention was 1 higher (1.9 lower to 4.2 higher)	101 (1 RCT)	⨁◯◯◯ VERY LOW ^d,e^
**Postoperative complications** (4 to 48 weeks)	Not estimable	-	Evidence of no difference between groups	829 (3 RCTs)	⨁◯◯◯ LOW ^d,f^
**Average length of stay **^**c**^ (1 to 12 weeks)	Not estimable	-	Evidence of no difference between groups	728 (2 RCTs)	⨁◯◯◯ VERY LOW ^f,g,h^

^a^The risk in the intervention group (and its 95% confidence interval) is based on the assumed risk in the comparison group and the relative effect of the intervention (and its 95% CI). *CI* Confidence interval, *HRQoL* Health-related quality of life, *MD* Mean Difference, *RR* Risk ratio

^b^EORTC QLQ-C30: higher scores indicate better functioning (scaled from 0 to 100)

^c^Administrative data: higher number of days indicate worse outcome

^d^Downgraded by two levels due to selection bias, no blinding of personnel (performance bias) and selective outcome reporting

^e^Downgraded by two levels due to small sample size and wide confidence intervals (imprecision)

^f^Downgraded by one level due to wide confidence intervals (imprecision)

^g^Downgraded by one level due to selection bias (allocation concealment)

^h^Downgraded by one level due to inconsistency (opposite results)

### Comparison 1: combined training vs rehabilitation/usual care

#### Health-related quality of life (HRQoL)

**Fig. 4:**
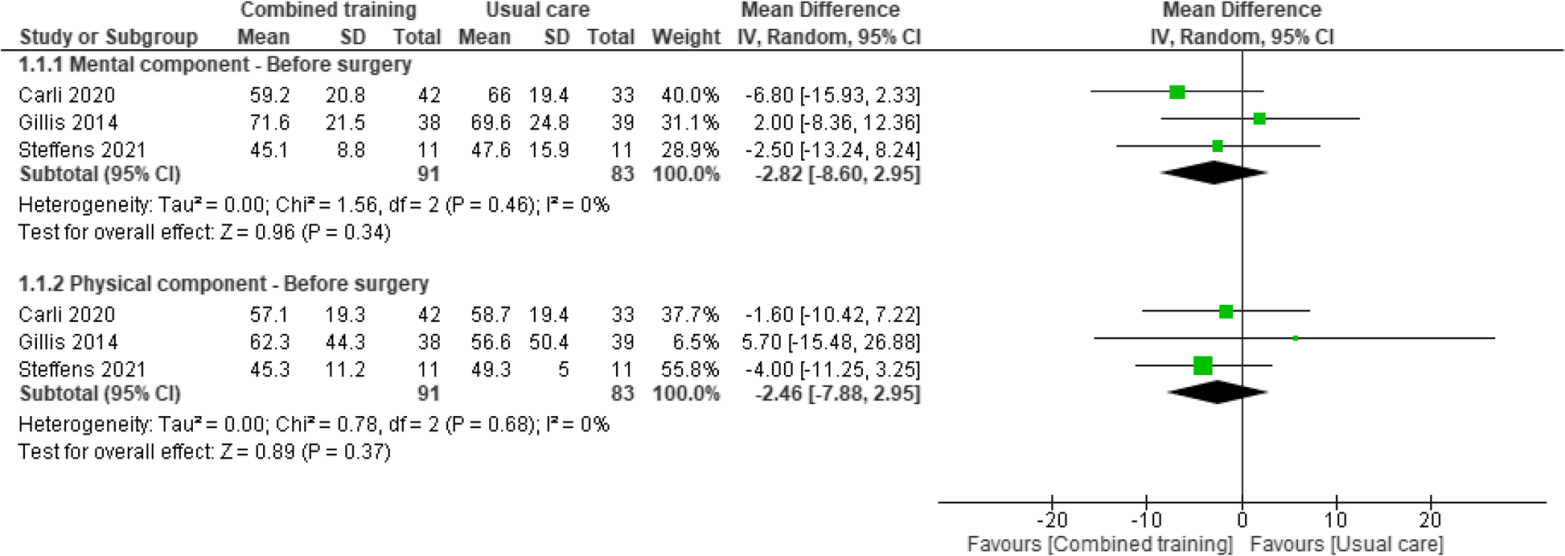
Effects of prehabilitation programs (with combined training) vs rehabilitation/usual care in HRQoL (physical and mental component) before surgery

Seven studies reported data before surgery (i.e., post-intervention) [32, 34, 43]. Pooled data from three studies (174 participants with colorectal cancer) [32, 34, 48] showed evidence of no difference between groups in the physical component (MD –2.46, 95% CI -7.88 to 2.95) or the mental health component of HRQoL (MD -2.82, 95%CI -8.60 to 2.95) (Fig. 4). Santa Mina 2018 [45] reported similar findings (Effect estimate 2.1, 95% CI –4.25 to 8.47). Sebio García 2017 [43] observed better HRQoL scores in the combined training group (mean change 4.5 points higher), yet outcome data for the control group were not reported. It is uncertain whether combined training improves HRQoL before surgery because the certainty of this evidence is very low (Table 3).

**Fig. 5:**
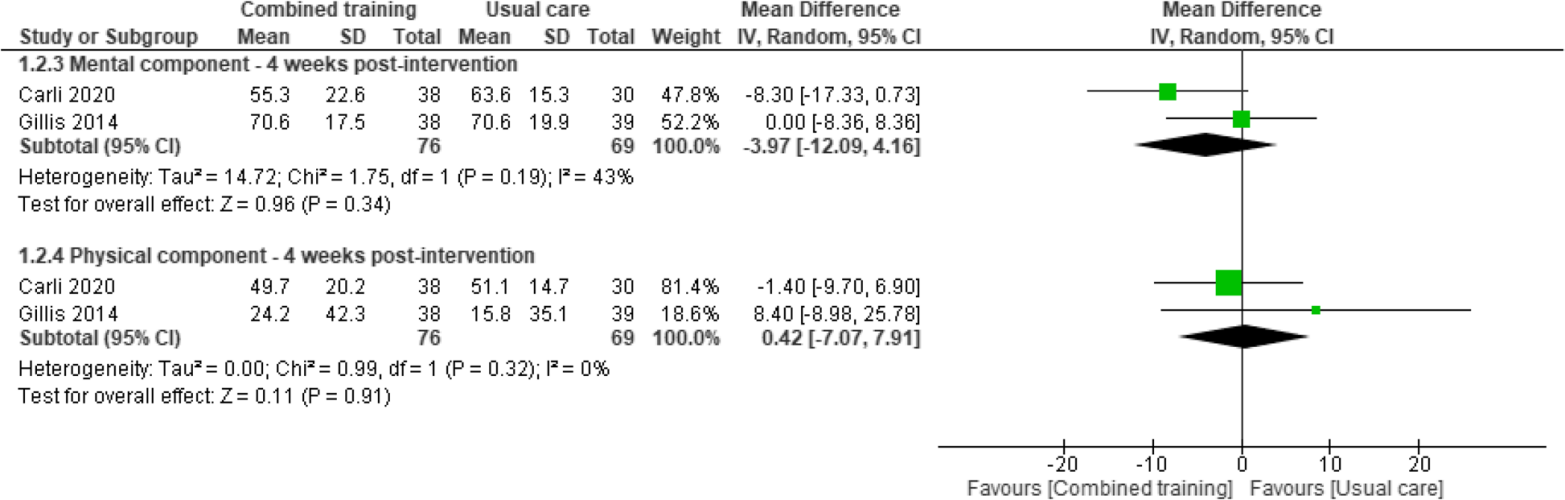
Effects of prehabilitation programs (combined training) vs rehabilitation/usual care in HRQoL (physical and mental component) at four weeks post-intervention

Sebio-García 2017 [43] provided post-operative data in 19 participants with lung cancer. The combined training group reported less decline in their HRQoL than usual care controls (MD prehabilitation -2.8, standard error 5.8 vs. MD usual care –7.4, standard error 5.3; p*-*value for the interaction effect 0.06). Further, pooled analysis from the two studies that reported outcome data at four weeks post-intervention (145 participants with colorectal cancer) [32, 34] showed a lack of evidence of an effect between groups in the physical component of HRQoL (SMD 0.42, 95%CI -7.07 to 7.91) or the mental component (SMD -3.97, 95%CI -12.09 to 4.16) (Fig. 5). Steffens 2021 [45] reported evidence of no effect of prehabilitation programs compared to usual care ten days after surgery.

Three studies reported long-term data [34, 43, 45]. Gillis 2014 [34] found evidence of no difference between combined training (mean score 74.3, SD 26.1) and usual care (mean score 72.3, SD 24.2) at eight weeks post-intervention. Similar findings were reported by Sebio-García 2017 [43] in participants with lung cancer, and by Santa Mina 2018 [45] in participants with prostate cancer at 12 weeks and at 26 weeks. Overall, these studies indicated a lack of evidence of an effect between groups in HRQoL in the long term. Therefore, it is uncertain whether combined training improves HRQoL in the long term because the certainty of the evidence is very low (Table 3).

#### Muscular strength

Four studies found evidence of no effect between groups in muscular strength before and after the surgery [13, 43, 45, 48]. Similar findings were reported at 12- and 26-weeks post-intervention in individuals with prostate cancer [45]. It is uncertain whether combined training improves muscular strength because the certainty of the evidence is very low (Table 3).

#### Postoperative complications

**Fig. 6:**
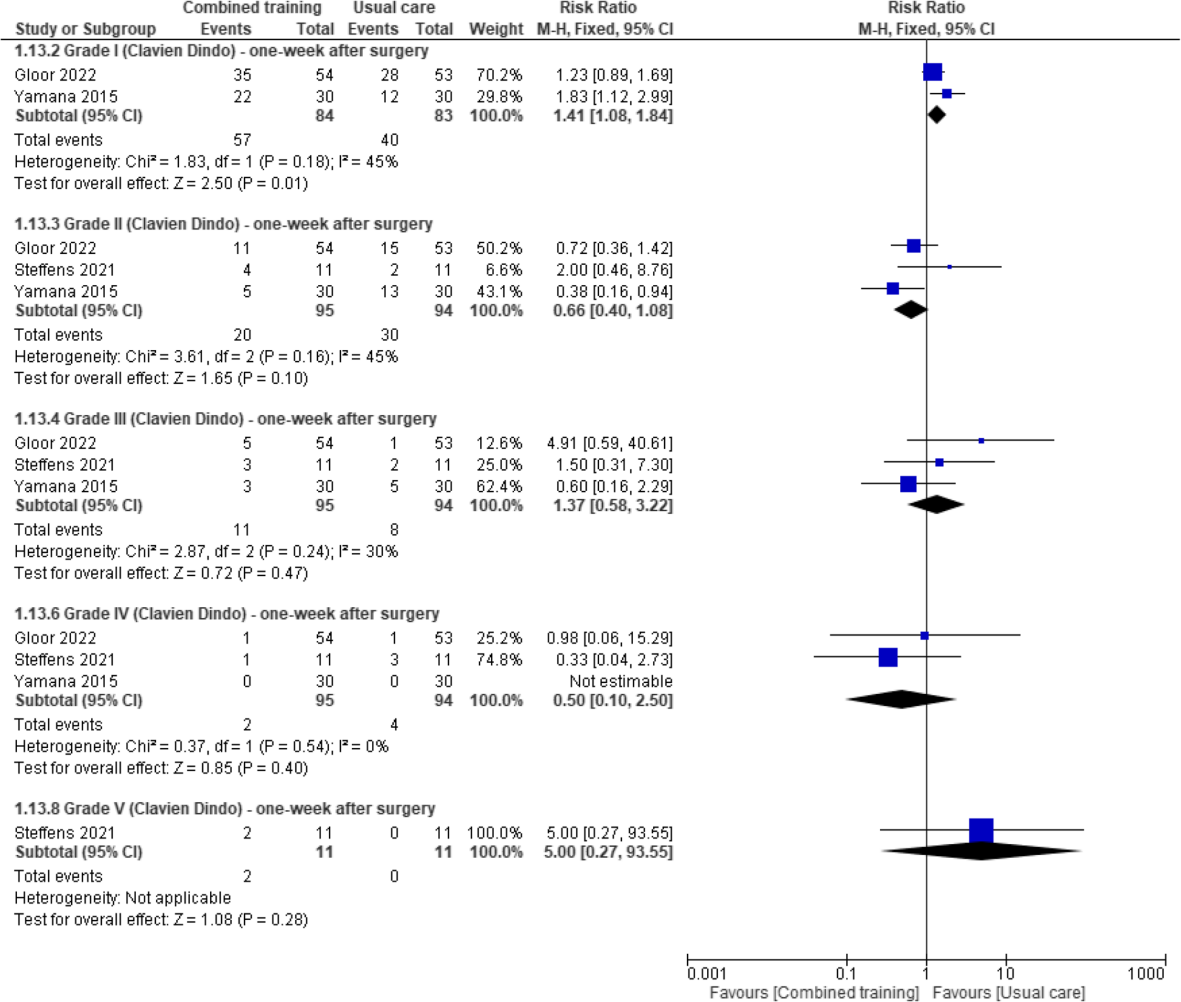
Effects of prehabilitation programs (with combined training) vs rehabilitation/usual care in postoperative complications (The Clavien-Dindo Classification: high grades indicate worse outcome) after surgery

Three studies reported on postoperative complications incidence one week after surgery [35, 48, 51]. Pooled data from two studies [35, 51] (97 participants with colorectal and esophageal cancer) found that prehabilitation programs with combined training increased the risk of incidence of grade I complications compared to usual care (RR 1.41, 95% CI 1.08 to 1.84) (Fig. 6). Gloor 2022 [34] and Yamana 2015 [50] found no grade V complications one week after surgery. Pooled analysis from three studies (189 participants with colorectal and esophageal cancer) showed no between-group differences on grade II complications (RR 0.66, 95% CI 0.40 to 1.08), grade III complications (RR 1.37, 95% CI 0.85 to 3.22), grade IV complications (RR 0.50, 95% CI 0.10 to 2.50), or grade V complications (RR 5.0, 95% CI 0.27 to 93.55) one week before surgery (Fig. 6).

**Fig. 7:**
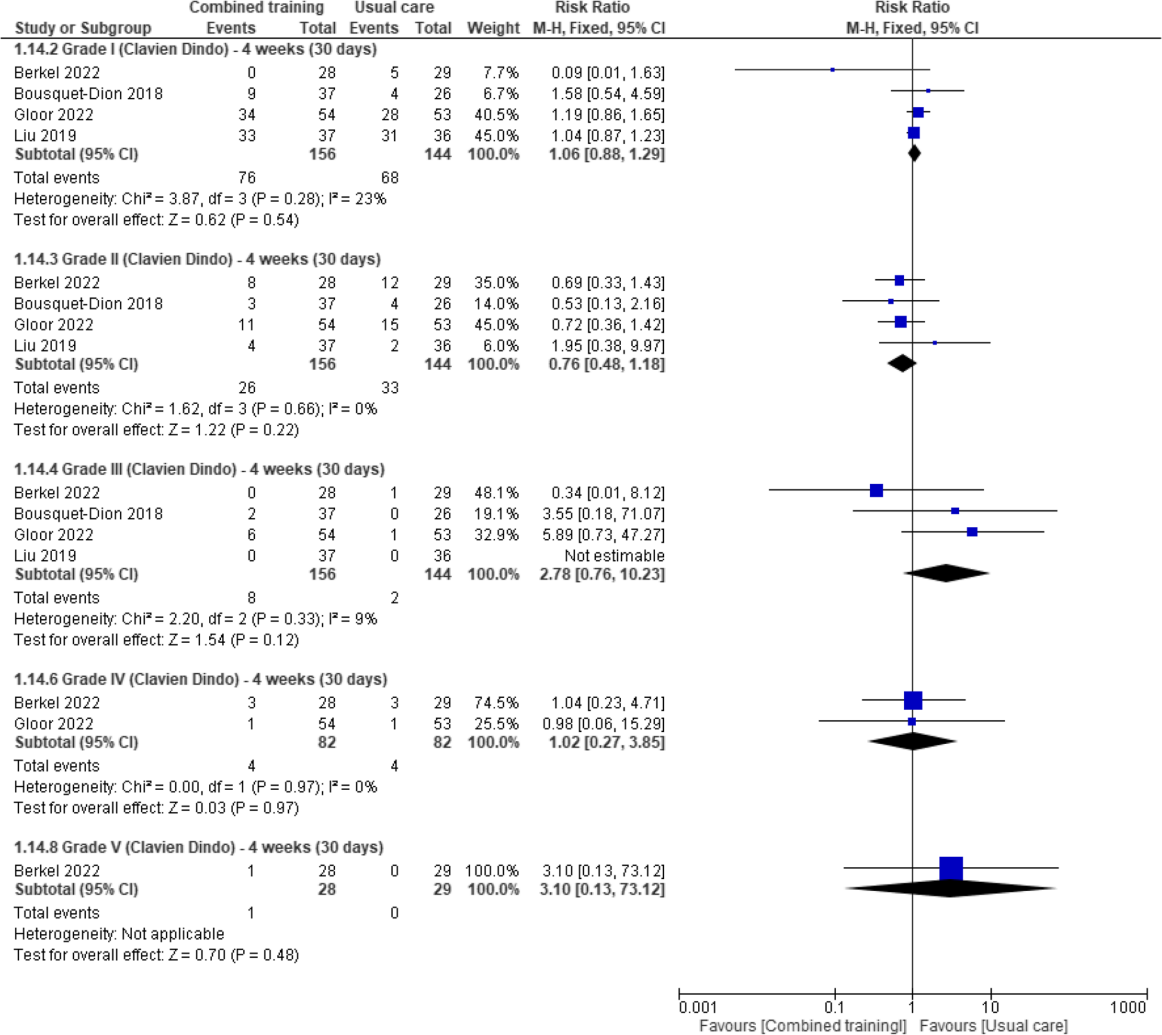
Effects of prehabilitation programs (with combined training) vs rehabilitation/usual care in postoperative complications (The Clavien-Dindo Classification: high grades indicate worse outcome) at four weeks (30 days)

Four weeks after surgery, five studies measured this outcome with the Clavien-Dindo classification [29, 30, 32, 35, 40] whereas Licker 2016 [39] used the modified version of the thoracic mortality and morbidity (TMM) classification system. Pooled data from four studies (300 participants with colorectal and lung cancer) found evidence of no difference between groups in the incidence of grade I complications (RR 1.06, 95% CI 0.88 to 1.29), grade II complications (RR 0.76, 95%CI 0.48 to 1.18), or grade III complications (RR 2.78, 95%CI 0.76 to 10.23) (Fig. 7). Pooled data from two studies [29, 35] (164 participants with colorectal cancer) provided the same evidence of no effect for grade IV (RR 1.02, 95%CI 0.27 to 3.85) and grade V complications (RR 3.10, 95%CI 0.13 to 73.12) four weeks after surgery (Fig. 7). Similar effects were reported by Licker 2017[39] using the Thoracic Morbidity and Mortality (TMM) system, and by Carli 2010 [31] with the 30-day Comprehensive Complications Index (adjusted mean difference –3.2, 95% CI –11.8 to 5.3; p = 0.45).

This evidence of no effect of combined training was confirmed by Gloor 2022 [35] six weeks after surgery (RR 1.47, 95% CI 0.56 to 3.85; n = 107 participants), by two studies eight- and nine weeks after surgery in individuals with colorectal cancer [31, 34], by Sebio Garcia 2017 [43] at 12 weeks, and finally by Santa Mina 2018 [45] 26 weeks after surgery. Overall, it is uncertain whether combined training reduces the incidence of postoperative complications because the certainty of the evidence is very low (Table 3).

#### Average length of stay (ALOS)

Four studies reported outcome data one-week after surgery (some reported this as at discharge) [13, 35, 36, 48]. Pooled data from three studies [35, 45] (148 participants with colorectal cancer) that compared prehabilitation programs with combined training versus usual care found no difference between groups in ALOS (MD -0.52 days, 95% CI -2.19 to 1.16 days). Similar findings were reported by Dronkers 2022 [13] after surgery (41 participants).

**Fig. 8:**
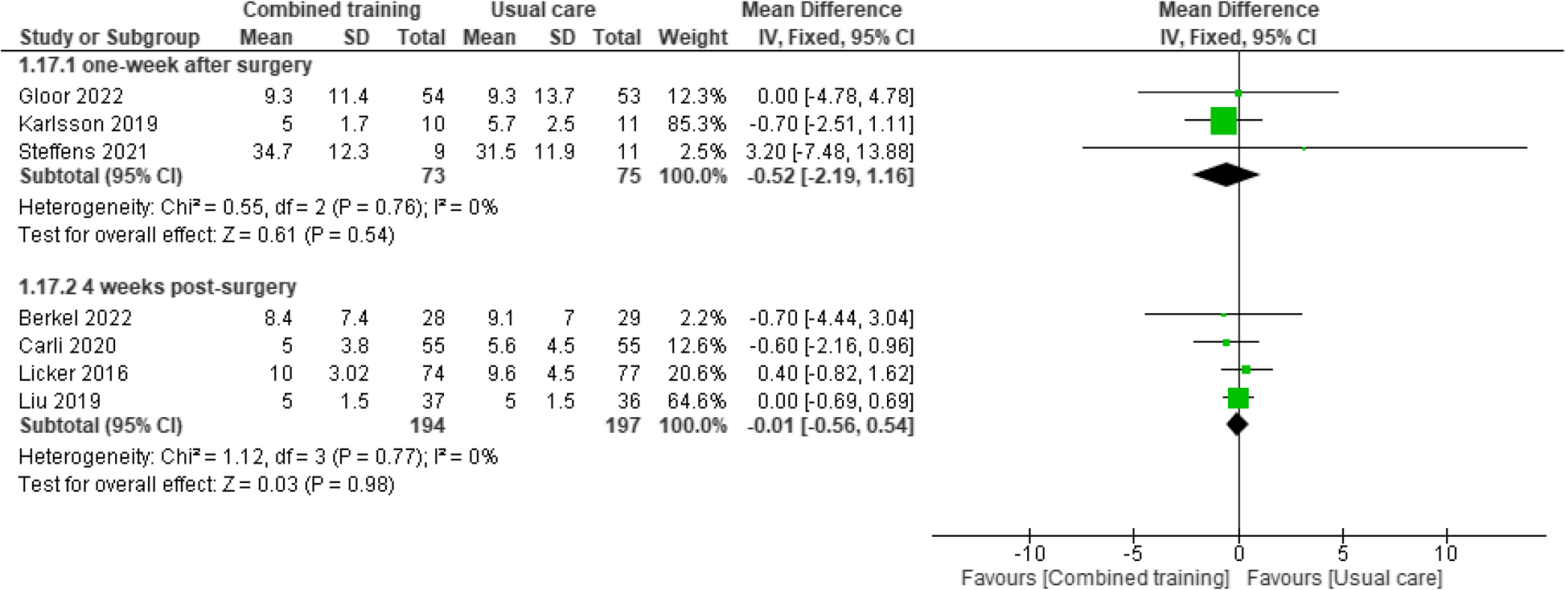
Effects of prehabilitation programs (with combined training) vs. usual care in average length of stay atone- and four weeks after surgery

Imputed data from four studies (391 participants with lung and colorectal cancer) [29, 32, 39, 40] provided evidence of no difference in effects between groups in ALOS four weeks after surgery (MD -0.01 days, 95% CI -0.56 to 0.54) (Fig. 8). Similar findings were reported for the remaining follow-up periods of eight weeks [34], nine weeks [31], 12 weeks [43], and 26 weeks [45] after surgery. Overall, it is uncertain whether prehabilitation programs with combined training reduce ALOS because the certainty of this evidence is very low (Table 3).

#### Handgrip strength

Santa Mina 2018 [45] found lack of evidence of an effect between groups in handgrip strength in 61 participants with prostate cancer up to 26 weeks post-intervention (difference at 26 weeks: 4.44, 95% CI 0.65 to 8.23). Peng 2021 [50] reported better handgrip strength in the prehabilitation group compared to usual care before surgery (30.1 ± 5.4 kg vs. 25.4 ± 4.9 kg on the day before surgery, P = 0.037) and 72 h after surgery (25.7 ± 4.8 kg vs. 22.3 ± 8.2 kg, P = 0.018). Overall, it is uncertain whether prehabilitation programs with combined training improve handgrip strength because the certainty of the evidence is very low (Table 3).

### Physical activity levels

#### Light physical activity

**Fig. 9:**
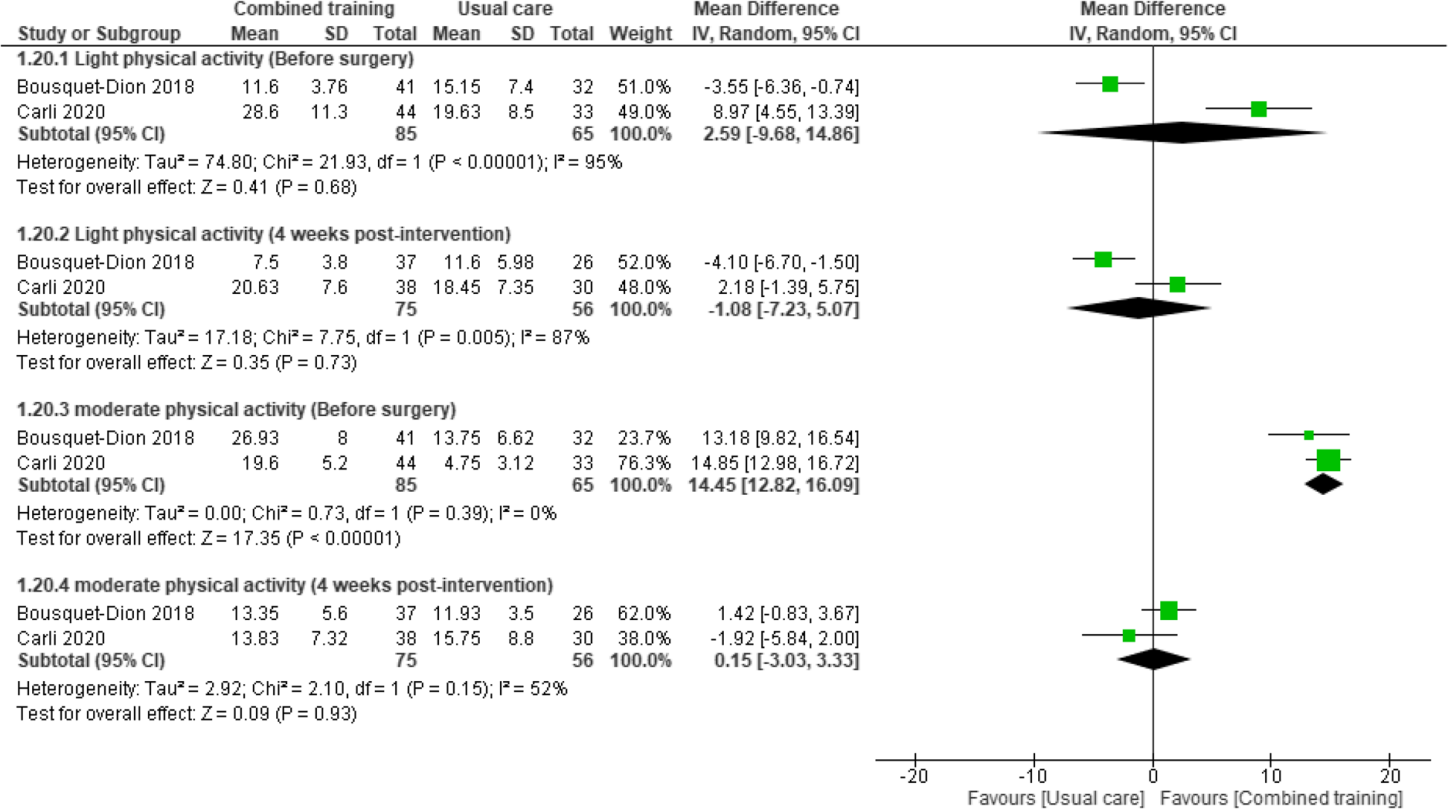
Effects of prehabilitation programs (with combined training) vs. rehabilitation/usual care in physical activity levels before- and four weeks after surgery

Five studies reported outcome data before surgery [30–32, 34, 45]. Pooled data from two studies [30, 32] showed evidence of no difference in effect between groups in light physical activity levels among 150 participants with colorectal cancer (MD 2.59 kcal/kg/wk, 95% CI -9.68 to 14.86). These findings were further confirmed by the remaining three studies before surgery [32, 34, 45], as well as by pooled data four weeks after surgery (MD -1.08, 95% CI -7.23 to 5.07). Based on this evidence, it is uncertain whether prehabilitation programs with combined training improve light physical activity levels because the certainty of the evidence is very low (Table 3 and Fig. 9).

#### Moderate physical activity

Four studies measured this outcome before surgery [13, 30, 32, 48]. Pooled data from two studies [30, 32] suggested that prehabilitation programs improved moderate physical activity levels compared to usual care before surgery (MD 14.45 kcal/kg/wk, 95% CI 12.82 to 16.09), but resulted in a lack evidence of between-group difference four weeks after surgery (MD 0.15 kcal/kg/wk, 95%CI -3.03 to 3.33). The remaining three studies reported similar findings at four-weeks [31, 34, 45], which were confirmed at 8 weeks [29, 33, 35, 37], 9 weeks [31], 12 weeks [45], and 26 weeks [45] post-intervention. In addition, two studies [13, 47] reported different units of physical activity and were therefore not entered into the meta-analysis. Both studies found evidence of no difference between groups before surgery in the Physical Activity Questionnaire (LAPAQ) activities (min/day) [13] or in the IPAQ [48] (Fig. 9).

All in all, it is uncertain whether prehabilitation programs with combined training improve moderate physical activity levels because the certainty of the evidence is very low (Table 3 and Fig. 9).

### Comparison 2: aerobic training (HIIT and MICT) vs usual care

#### Health-related quality of life (HRQoL)

Dunne 201 [33] found that a prehabilitation program with HIIT improved HRQoL compared to usual care four weeks after surgery in individuals undergoing elective liver resection for colorectal liver metastases (MD in SF-36 total scores 11 points, 95% CI 1 to 21). Conversely, Moug 2019 [37] reported evidence of no effect between prehabilitation (telephone-guided walking program) and usual care 12 weeks after surgery in 39 participants with rectal cancer undergoing neo-adjuvant chemoradiotherapy (MD in FACT-C total score 0.9 points, 95% CI -6.2 to 8.0).

#### Muscular strength

Moug 2019 [37] found evidence of no effect between prehabilitation (telephone-guided walking program) and usual care 12 weeks after surgery (MD sit-to-stand test, no. completed in 30 secs -0.6, 95% CI -3.3 to 2.2).

#### Postoperative complications

**Fig. 10:**
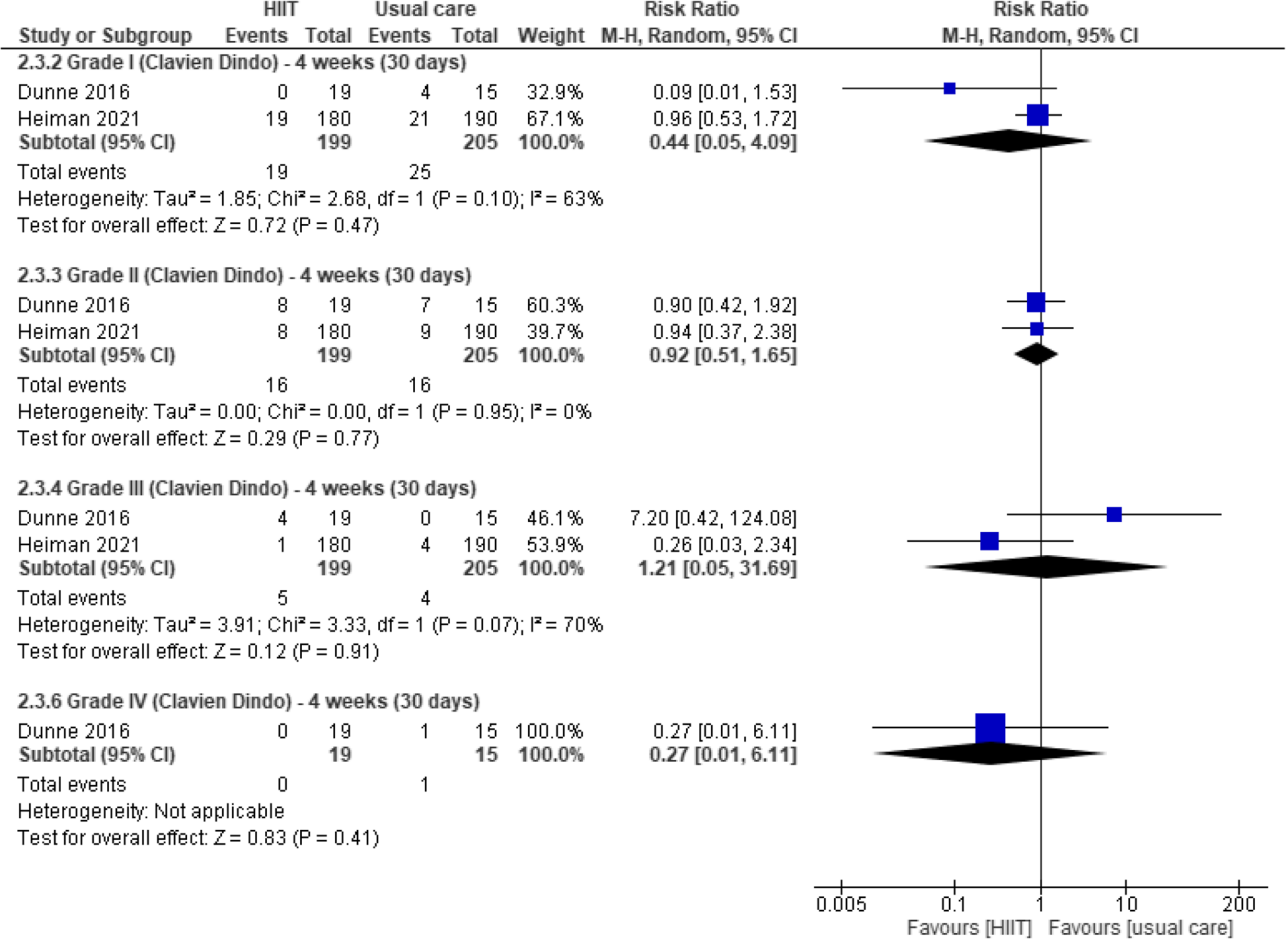
Effects of aerobic training (HIIT and MICT) vs usual care in postoperative complications (The Clavien-Dindo Classification: high grades indicate worse outcome) at four weeks (30 days)

Three studies measured this outcome four weeks after surgery [33, 47, 49]. Pooled data from two studies (405 participants with colorectal, bladder, and breast cancer) [33, 49] provided evidence of no difference between groups in the incidence of grade I complications (RR 0.44, 95% CI 0.05 to 4.09), grade II complications (RR 0.92, 95% CI 0.51 to 1.65), grade III complications (RR 1.21, 95% CI 0.05 to 31.69) or grade IV complications (RR 0.27, 95% CI 0.01 to 6.11) (Fig. 10). At the same timepoint, Banerjee 2018 [47] and Dunne 2016 [33] found no between-group differences in Clavien-Dindo grade ≥ 1. This lack of effect was confirmed by Moug 2019 [37] and Heiman 2021 [49] at 12-week follow-up.

#### Average length of stay (ALOS)

**Fig. 11:**
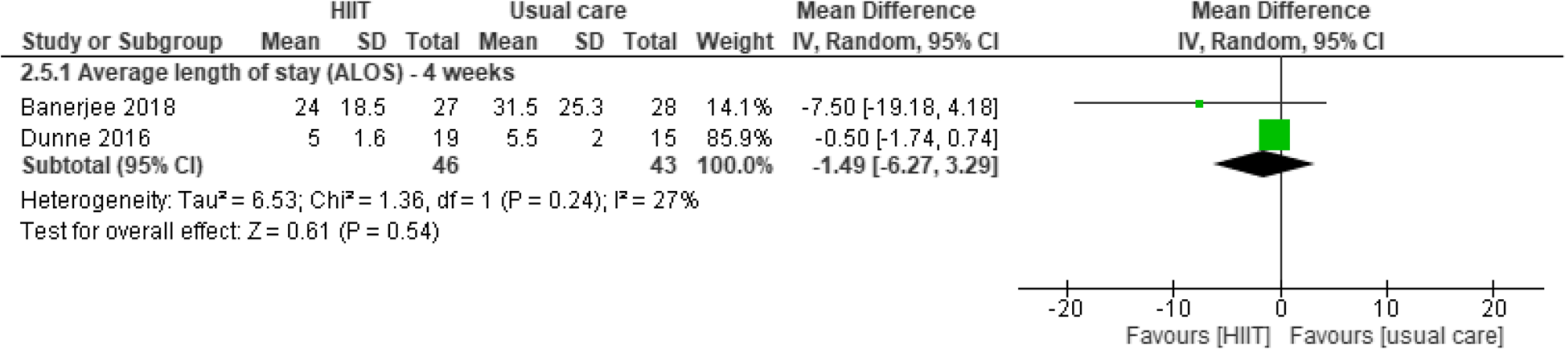
Effects of aerobic training (HIIT and MICT) vs usual care in average length of stay four weeks after surgery

Moug 2019 [37] found no between-group differences in ALOS one week after surgery. Three studies reported outcome data at four weeks after surgery [33, 47, 49]. Pooled data from two studies [33, 47] (89 participants with colorectal and bladder cancer) provided evidence of no difference between prehabilitation programs with HIIT and usual care in ALOS (MD -1.49 days, 95%CI -6.27 to 3.29). These findings were confirmed by Heiman 2021 [49] four weeks after surgery Fig. 11.

#### Physical activity levels

Moug 2019 [37] found evidence of no effect on the time spent being active between study groups 12 weeks after surgery (group difference 0.3%, 95% CI -1.7 to 2.2). Similar findings were reported by Heiman 2021 [49] using the Saltin–Grimby Physical Activity Level Scale (before versus after surgery).

Overall, it is uncertain whether aerobic training (HIIT or MCIT) has an effect on HRQoL, muscular strength, postoperative complications, ALOS, and physical activity levels (very-low certainty of evidence) (Table 4).

### Comparison 3: respiratory muscle training plus aerobic training vs usual care

Three studies reported data for this comparison in 161 participants with lung cancer [38, 41] as follows:

#### Health-related quality of life (HRQoL)

Lai 2017 [42] found evidence of no difference in effect between groups in HRQoL four weeks after surgery (MD 1.1, 95% CI -1.9 to 4.2).

#### Postoperative complications

Lai 2017 [42] found evidence of no difference between groups in the incidence of postoperative complications within 30 days after surgery (RR 0.35, 95% CI 0.14 to 0.90). Similar findings were reported by Pehlivan 2011 [41] (RR 0.2, 95% CI 0.02 to 1.6). The studies did not report the tool used to measure outcome data. This lack of an effect was also confirmed by Onerup 2022 [38] using the Clavien-Dindo classification four, 12, and 48 weeks after surgery.

#### Average length of stay (ALOS)

Pehlivan 2011 [41] reported evidence of no difference between groups in ALOS one week after surgery (MD -4.2 days, 95%CI -5.7 to -2.7). Similar findings were reported by Onerup 2022 [38] up to 90 days postoperatively.

Overall, it is uncertain whether respiratory muscle training plus aerobic training has an effect on HRQoL, postoperative complications, and ALOS because the certainty in this evidence is very low (Table 5).

### Comparison 4: respiratory muscle training plus resistance training vs usual care

#### Health-related quality of life (HRQoL)

Peng 2021 [50] used the 40-items QOR-40, which provided a total score and sub scores in five dimensions, namely patient support, comfort, emotions, life ability, and physical well-being, and pain. The study found no between-group differences before surgery but reported better sub scores for life ability and physical well-being in the prehabilitation group relative to usual care 72 h after surgery (19.6 ± 3.1 vs. 15.7 ± 2.8, P = 0.032 and 43.4 ± 5.3 vs. 39.2 ± 6.1, P = 0.029, respectively). No differences were noted in five dimensions 30 days after surgery.

#### Postoperative complications

Peng 2021 [50] found evidence of no between-group differences in the incidence of bowel-related and non-bowel related- adverse events at 30-day follow-up. Adverse event rates varied between 5.8% and 10.8%.

#### Average length of stay (ALOS)

In Peng 2021 [50], the total length of hospital stay after surgery was 58.4 h (interquartile range [IQR]: 41.6–69.8) and 62.5 h (IQR: 43–73.4) in the prehabilitation and usual care groups, respectively (P = 0.061).

#### Handgrip strength

Peng 2021 [50] found the dominant hand grip in the prehabilitation group to be stronger than that in the usual care group on the day before surgery (25.7 ± 4.8 kg vs. 22.3 ± 8.2 kg, P = 0.018).

Overall, our certainty in the evidence base for this comparison was rated as low due to methodological limitations (unblinded intervention providers) and imprecise results (small sample size). Respiratory muscle training plus resistance training may result in little to no difference in the abovementioned outcomes.

### Comparison 5: pelvic floor training vs usual care

Three studies provided outcome data for HRQoL in individuals with prostate cancer [28, 44, 46]. None of the studies reported evidence of an effect in this outcome between groups across the follow-up periods. Centemero 2010 [44] and Laurienzo 2013 [28] found evidence of no effect in HRQoL four weeks post-intervention. Ocampo-Trujillo 2014 [46] reported similar findings at eight weeks post-intervention. Finally, Centemero 2010 [44] and Laurienzo 2013 [28, 44] confirmed these findings up to 24 weeks after surgery. It is uncertain whether pelvic floor training improves HRQoL because the certainty of this evidence is very low. We downgraded our certainty in the evidence due to methodological limitations (random sequence generation, unblinded intervention providers, and selective outcome reporting) and imprecise results (small sample size and wide confidence intervals).

## Discussion

### Summary of main results

This systematic review synthesized evidence from 25 RCTs on the benefits and harms of prehabilitation programs compared with usual care for individuals with cancer. The risk of bias across the studies showed no major concerns regarding randomization, but some concerns were identified for outcome measurement, selective outcome reporting, and attrition bias. The studies provided outcome data for five comparisons with usual care (rehabilitation): combined training, aerobic training, respiratory muscle training plus aerobic training, respiratory muscle training plus resistance training, and pelvic floor training.

Data from 14 studies suggested that prehabilitation programs of combined training confer little to no effect on HRQoL, muscular strength, postoperative complications, ALOS, handgrip strength, or physical activity levels compared to rehabilitation or usual care. Three small studies evaluated the effects of HIIT as part of a prehabilitation program but found no evidence of an effect on HRQoL or postoperative complications. This lack of evidence of an effect on those outcomes was also observed for prehabilitation programs combining respiratory muscle training and aerobic training. Finally, three studies provided evidence of no difference between prehabilitation programs of pelvic floor training and usual care in HRQoL.

### Overall completeness and applicability

The knowledge base of this systematic review mostly applies to individuals aged 60 to 70, with colorectal, prostate, and lung cancer. Few studies provided sociodemographic data. Besides, the studies failed to provide information about key aspects of the prehabilitation program's implementation, such as rules for starting levels and program progression. This poses a challenge to the context-specific application of our findings.

To date, the 25 studies included in this systematic review are not sufficient to fully address our primary objective. However, we believe that the 14 studies that compared combined training to rehabilitation or usual care offer a consistent evidence base of the lack of effect for this comparison. Conversely, a few unpowered studies provide no clear evidence for the remaining comparisons. Hence, it is likely that new, well-conducted studies will substantially change our findings.

### Certainty of the evidence

Very low-certainty evidence underpinned all the comparisons in this systematic review. The included studies evaluated different modes of prehabilitation programs, where combined training (i.e., aerobic and resistance training) was the main comparison. In some studies, we found scarce information about the characteristics of the participants, the implementation of the prehabilitation programs, and poor reporting of outcome data. Our certainty in the evidence was further downgraded due to the studies’ risk of bias, such as a lack of allocation concealment and blinding of participants and providers and selective outcome reporting. The lack of blinding of participants and intervention providers can lead to an overestimation of the effect estimate [61, 62]. Blinding participants in exercise training trials is not easy [63], and it is likely that participants who are aware of their intervention may differ from blinded participants in how they report outcomes or on their performance in the study [64]. These aspects become more relevant in the context of this systematic review given the large number of self-reported outcomes across studies. Furthermore, most of the studies had a small number of participants, wide confidence intervals, and high heterogeneity in the effects across them. Undertaking a sensitivity analysis to explore these limitations was not appropriate due to the small number of studies, which could bias any effect estimate.

### Strengths and limitations

The strengths of this systematic review were the use of validated and transparent methods to assess the reporting of the exercise interventions within the prehabilitation programs and the certainty of the evidence. Likewise, our outcome prioritization process represents another strength, although this was reliant on what the previous reviews had reported. The integration of these processes enhances the use of this review in evidence-informed decision-making scenarios. Regarding the limitations, our search strategy was not peer-reviewed, and we believe that the comparisons in this review may be biased due to the incomplete reporting of intervention-related domains. We contacted the authors for further details, but the response rate was low. In an effort to tackle this limitation, we searched clinical trial registries to detect unpublished trials. Other approaches for assessing selective outcome reporting, such as funnel plots and statistical tests, were deemed inappropriate because of the small number of studies included in each comparison.

### Agreements and disagreements with other reviews

Several systematic reviews have explored the effects of prehabilitation programs for individuals with cancer on various outcome measures [65–77]. The methods used in those reviews varied considerably, as did their findings. Our findings of very low certainty in the effects of prehabilitation programs on HRQoL align with those reported by eight systematic reviews published recently [65, 66, 68, 73–75, 78, 79]. Contradictory findings in different HRQoL domains have been reported by other systematic reviews [67, 72, 77, 80, 81]. Similarly, our findings of very low certainty on postoperative complications coincide with those of low certainty reported by Xiang Li and colleagues in 2016 [76]. Other systematic reviews have reported significant reductions in this outcome [66–68, 70–73, 77, 81, 82]. A similar trend is observed in the literature about other outcome measures in this review.

Several factors, including differences in baseline characteristics of the participants and program implementation explain the discrepancies between our findings and those of other systematic reviews. Besides, the lack of an assessment of the certainty of the evidence has an impact on the review findings, and the use of different approaches to conduct this assessment influences authors’ judgments about the body of evidence [83]. It is worth noting that the restriction of exercise training as the main component of prehabilitation programs in our review did not explain the discrepancies with other systematic reviews, as our findings are similar to those reported by reviews that included prehabilitation programs of exercise training plus dietary counseling or other multimodal approaches [81, 82, 84].

### Implications for practice and research

The findings of this systematic review emphasize the need for further well-conducted RCTs to better inform recommendations for cancer prehabilitation programs. The very low certainty of the evidence about the benefits and harms of prehabilitation programs constraints the use of our findings in decision-making scenarios (e.g., clinical practice guidelines). The lack of safety data and the use of heterogeneous tools for outcome measurement pose additional limitations. In order to strengthen the certainty of the evidence, further studies may benefit from addressing the gaps identified in our assessment of the prehabilitation programs reporting, and should adhere to reporting checklists, such as CONSORT [85] and CERT [24]. Besides, further studies may benefit from the use of core outcome sets (COMET initiative [75]) and cross-center collaborations.

## Conclusion

Prehabilitation programs focusing on exercise training may have an effect on adults with cancer, but the evidence is very uncertain. Further well-conducted randomized controlled trials are still required to test the role of exercise training as the main component of prehabilitation programs. We assessed the certainty of the body of evidence as very low due to serious study limitations, imprecision, and indirectness. Further research is needed before we can draw a more certain conclusion.

### Supplementary Information

**Additional file 1.** PRISMA 2020 checklist.

**Additional file 2.** Search strategies.

**Additional file 3.** Excluded studies at full text.

**Additional file 4.** Characteristics of the ongoing studies.

**Additional file 5.** Characteristics of the prehabilitation programs (*n* = 25).

**Additional file 6.** Subgroup analysis for the completeness of reporting of the exercise training interventions in the prehabilitation programs.

**Additional file 7.** Other outcome measures reported in the included studies.

## Data Availability

Not applicable.

## Abbreviations

ALOS: Average length of stay
CI: Confidence interval
CERT: Consensus on Exercise Reporting Template
CHAMPS: The Community Healthy Activities Model Program for Seniors
Con: Control
EORTC QLQ-C30: The European Organization for Research and Treatment of Cancer Quality of Life Questionnaire Core 30
FACT-P: The Functional Assessment of Cancer Therapy-Prostate
GRADE: Grading of Recommendations, Assessments, Development and Evaluation
HRQoL: Health-related quality of life
HIIT: High-intensity interval training
HRR: Heart rate reserve
ICC: The Comprehensive Complication Inde
ICS SF: The International Continence Society male Short Form
Int: Intervention
IQR: Interquartile range
MHR: Maximum heart rate
MD: Mean difference
MCS: Mental component summary
N: Number of participants analyzed in the study
NR: Not reported
PRISMA-P: Preferred Reporting Items for Systematic Review and Meta-Analyses Protocol
PROSPERO: Prospective Register of Systematic Reviews
PCS: Physical component summary
RM: Repetition maximum
RR: Risk ratio
SF-36: The Short Form-36 Health Survey
SF-36 v2: The Short Form-36 Health Survey version 2
SD: Standard deviation
SE: Standard error
SMD: Standardized mean difference
The Clavien-Dindo: Classification for surgical complications
TMM: Thoracic morbidity and mortality
WR: Work rate
W: Workload
