# Supplementary material for: Prehabilitation programs for individuals with cancer: a systematic review of randomized-controlled trials
Source: Syst Rev. 2023 Nov 17;12:219. doi: 10.1186/s13643-023-02373-4 (PMC10655304; doi:10.1186/s13643-023-02373-4)
Supplement: Supplementary file 2 — Additional file 2. Search strategies. [file 13643_2023_2373_MOESM2_ESM.docx]

# **Additional file 2.** **Search strategies**

## **Cochrane Central Register of Controlled Trials**

1 exp neoplasms/

2 neoplasms.tw.

3 exp lymphoma/

4 lymphoma.tw.

5 lymphoma.tw.

6 exp radiotherapy/

7 radiotherapy.tw.

8 exp "Bone Marrow Transplantation"/

9 Bone Marrow Transplantation.tw.

10 exp cancer/

11 cancer.tw.

12 leukaemia.mp. [mp=ti, ot, ab, tx, kw, ct, sh, fx, hw]

13 leukaemia.tw.

14 exp tumour/

15 tumour.tw.

16 exp tumor/

17 tumor.tw.

18 malignan.mp. [mp=ti, ot, ab, tx, kw, ct, sh, fx, hw]

19 malignan.tw.

20 carcino.mp. [mp=ti, ot, ab, tx, kw, ct, sh, fx, hw]

21 carcino.tw.

22 exp adenocarcinoma/

23 adenocarcinoma.tw.

24 neutropeni.mp. [mp=ti, ot, ab, tx, kw, ct, sh, fx, hw]

25 1 or 2 or 3 or 4 or 5 or 6 or 7 or 8 or 9 or 10 or 11 or 12 or 13 or 14 or 15 or 16 or 17 or 18 or 19 or 20 or 21 or 22 or 23 or 24

26 exp prehabilitation/

27 prehabilitation.tw.

28 preoperative.mp. [mp=ti, ot, ab, tx, kw, ct, sh, fx, hw]

29 preoperative.tw.

30 pre-operative.mp. [mp=ti, ot, ab, tx, kw, ct, sh, fx, hw]

31 pre-operative.tw.

32 presurg.mp. [mp=ti, ot, ab, tx, kw, ct, sh, fx, hw]

33 presurg.tw.

34 pre-surg.mp. [mp=ti, ot, ab, tx, kw, ct, sh, fx, hw]

35 pre-surg.tw.

36 before surg.mp. [mp=ti, ot, ab, tx, kw, ct, sh, fx, hw]

37 before surg.tw.

38 before operat.mp. [mp=ti, ot, ab, tx, kw, ct, sh, fx, hw]

39 before operat.mp. [mp=ti, ot, ab, tx, kw, ct, sh, fx, hw]

40 before operat.tw.

41 exp rehabilitation/

42 rehabilitation.tw.

43 rehabilitat.mp. [mp=ti, ot, ab, tx, kw, ct, sh, fx, hw]

44 rehabilitat.tw.

45 physiotherap.mp. [mp=ti, ot, ab, tx, kw, ct, sh, fx, hw]

46 physiotherap.tw.

47 physical therap.mp. [mp=ti, ot, ab, tx, kw, ct, sh, fx, hw]

48 physical therap.tw.

49 26 or 27 or 28 or 29 or 30 or 31 or 32 or 33 or 34 or 35 or 36 or 37 or 38 or 39 or 40 or 41 or 42 or 43 or 44 or 45 or 46 or 47 or 48

50 exp exercise/

51 exercise.tw.

52 exercis.mp. [mp=ti, ot, ab, tx, kw, ct, sh, fx, hw]

53 exercis.tw.

54 aerobic.mp. [mp=ti, ot, ab, tx, kw, ct, sh, fx, hw]

55 aerobic.tw.

56 aerobic.tw.

57 endurance.mp. [mp=ti, ot, ab, tx, kw, ct, sh, fx, hw]

58 endurance.tw.

59 exp treadmill/

60 treadmill.tw.

61 exp walking/

62 walking.tw.

63 walk.mp. [mp=ti, ot, ab, tx, kw, ct, sh, fx, hw]

64 walk.tw.

65 exp "breathing exercises"/

66 breathing exercises.tw.

67 exp "respiratory muscle training"/

68 exp "respiratory muscle training"/

69 respiratory muscle training.tw.

70 bicycl.mp. [mp=ti, ot, ab, tx, kw, ct, sh, fx, hw]

71 cycling.tw.

72 50 or 51 or 52 or 53 or 54 or 55 or 56 or 57 or 58 or 59 or 60 or 61 or 62 or 63 or 64 or 65 or 66 or 67 or 68 or 69 or 70 or 71

73 25 and 49 and 72

74 limit 73 to yr="2020 - 2022"

## **MEDLINE (via PUBMED)**

1 neoplasms[Mesh]

2 neoplasms[tiab]

3 lymphoma[Mesh]

4 lymphoma[tiab]

5 lymphoma[tiab]

6 radiotherapy[Mesh]

7 radiotherapy[tiab]

8 Bone Marrow Transplantation[Mesh]

9 Bone Marrow Transplantation[tiab]

10 cancer[Mesh]

11 cancer[tiab]

12 leukaemia[tw]

13 leukaemia[tiab]

14 tumour[Mesh]

15 tumour[tiab]

16 tumor[Mesh]

17 tumor[tiab]

18 malignan[tw]

19 malignan[tiab]

20 carcino[tw]

21 carcino[tiab]

22 adenocarcinoma[Mesh]

23 adenocarcinoma[tiab]

24 neutropeni[tw]

25 1 OR 2 OR 3 OR 4 OR 5 OR 6 OR 7 OR 8 OR 9 OR 10 OR 11 OR 12 OR 13 OR 14 OR 15 OR 16 OR 17 OR 18 OR 19 OR 20 OR 21 OR 22 OR 23 OR 24

26 prehabilitation[Mesh]

27 prehabilitation[tiab]

28 preoperative[tw]

29 preoperative[tiab]

30 pre-operative[tw]

31 pre-operative[tiab]

32 presurg[tw]

33 presurg[tiab]

34 pre-surg[tw]

35 pre-surg[tiab]

36 before surg[tw]

37 before surg[tiab]

38 before operat[tw]

39 before operat[tw]

40 before operat[tiab]

41 rehabilitation[Mesh]

42 rehabilitation[tiab]

43 rehabilitat[tw]

44 rehabilitat[tiab]

45 physiotherap[tw]

46 physiotherap[tiab]

47 physical therap[tw]

48 physical therap[tiab]

49 26 OR 27 OR 28 OR 29 OR 30 OR 31 OR 32 OR 33 OR 34 OR 35 OR 36 OR 37 OR 38 OR 39 OR 40 OR 41 OR 42 OR 43 OR 44 OR 45 OR 46 OR 47 OR 48

50 exercise[Mesh]

51 exercise[tiab]

52 exercis[tw]

53 exercis[tiab]

54 aerobic[tw]

55 aerobic[tiab]

56 aerobic[tiab]

57 endurance[tw]

58 endurance[tiab]

59 treadmill[Mesh]

60 treadmill[tiab]

61 walking[Mesh]

62 walking[tiab]

63 walk[tw]

64 walk[tiab]

65 breathing exercises[Mesh]

66 breathing exercises[tiab]

67 respiratory muscle training[Mesh]

68 respiratory muscle training[Mesh]

69 respiratory muscle training[tiab]

70 bicycl[tw]

71 cycling[tiab]

72 50 OR 51 OR 52 OR 53 OR 54 OR 55 OR 56 OR 57 OR 58 OR 59 OR 60 OR 61 OR 62 OR 63 OR 64 OR 65 OR 66 OR 67 OR 68 OR 69 OR 70 OR 71

73 25 AND 49 AND 72

74 73 AND From 2020-2022

## **Embase (Elsevier)**

1 neoplasms/exp

2 neoplasms:ti,ab

3 lymphoma/exp

4 lymphoma:ti,ab

5 lymphoma:ti,ab

6 radiotherapy/exp

7 radiotherapy:ti,ab

8 'Bone Marrow Transplantation'/exp

9 'Bone Marrow Transplantation':ti,ab

10 cancer/exp

11 cancer:ti,ab

12 leukaemia

13 leukaemia:ti,ab

14 tumour/exp

15 tumour:ti,ab

16 tumor/exp

17 tumor:ti,ab

18 malignan

19 malignan:ti,ab

20 carcino

21 carcino:ti,ab

22 adenocarcinoma/exp

23 adenocarcinoma:ti,ab

24 neutropeni

25 #1 OR #2 OR #3 OR #4 OR #5 OR #6 OR #7 OR #8 OR #9 OR #10 OR #11 OR #12 OR #13 OR #14 OR #15 OR #16 OR #17 OR #18 OR #19 OR #20 OR #21 OR #22 OR #23 OR #24

26 prehabilitation/exp

27 prehabilitation:ti,ab

28 preoperative

29 preoperative:ti,ab

30 pre-operative

31 pre-operative:ti,ab

32 presurg

33 presurg:ti,ab

34 pre-surg

35 pre-surg:ti,ab

36 'before surg'

37 'before surg':ti,ab

38 'before operat'

39 'before operat'

40 'before operat':ti,ab

41 rehabilitation/exp

42 rehabilitation:ti,ab

43 rehabilitat

44 rehabilitat:ti,ab

45 physiotherap

46 physiotherap:ti,ab

47 'physical therap'

48 'physical therap':ti,ab

49 #26 OR #27 OR #28 OR #29 OR #30 OR #31 OR #32 OR #33 OR #34 OR #35 OR #36OR #37 OR #38 OR #39 OR #40 OR #41 OR #42 OR #43 OR #44 OR #45 OR #46 OR #47 OR #48

50 exercise/exp

51 exercise:ti,ab

52 exercis

53 exercis:ti,ab

54 aerobic

55 aerobic:ti,ab

56 aerobic:ti,ab

57 endurance

58 endurance:ti,ab

59 treadmill/exp

60 treadmill:ti,ab

61 walking/exp

62 walking:ti,ab

63 walk

64 walk:ti,ab

65 'breathing exercises'/exp

66 'breathing exercises':ti,ab

67 'respiratory muscle training'/exp

68 'respiratory muscle training'/exp

69 'respiratory muscle training':ti,ab

70 bicycl

71 cycling:ti,ab

72 #50 OR #51 OR #52 OR #53 OR #54 OR #55 OR #56 OR #57 OR #58 OR #59 OR #60 OR #61 OR #62 OR #63 OR #64 OR #65 OR #66 OR #67 OR #68 OR #69 OR #70 OR #71

73 #25 AND #49 AND #72

74 #73 AND [2020-2022]/py

## **ClinicalTrials.gov (http://clinicaltrials.gov)**

Terms searched cancer and prehabilitation

**Search Date:** June 10, 2022

**Condition or disease:** Cancer

**Other terms:** Prehabilitation

**Study type:** Interventional Studies (Clinical trials)

**Study Results:** All Studies

## **WHO International Clinical Trials Registry Platform (**<https://trialsearch.who.int/Default.aspx>**)**

Terms searched cancer and prehabilitation

**Search Date:** June 10, 2022

**in the Condition:** Cancer

**in the Intervention:** Prehabilitation
