# Supplementary material for: Prehabilitation programs for individuals with cancer: a systematic review of randomized-controlled trials
Source: Syst Rev. 2023 Nov 17;12:219. doi: 10.1186/s13643-023-02373-4 (PMC10655304; doi:10.1186/s13643-023-02373-4)
Supplement: Supplementary file 3 — Additional file 3. Excluded studies at full text. [file 13643_2023_2373_MOESM3_ESM.docx]

**Additional file 3.** Excluded studies at full-text

| **Excluded study** | **Reason for exclusion** |
| --- | --- |
| **Not a prehabilitation program (n=51)** | |
| Adams 2020 (1) | Not a prehabilitation program |
| Allen 2022 (2) | Not a prehabilitation program |
| Ausania 2019 (3) | Not a prehabilitation program |
| Barassi 2018 (7) | Not a prehabilitation program |
| Barberan-Garcia 2020 (9) | Not a prehabilitation program |
| Baré 2020 (10) | Not a prehabilitation program |
| Bausys 2020 (11) | Not a prehabilitation program |
| Berger-Richardson 2020 (13) | Not a prehabilitation program |
| Burgio 2006 (17) | Not a prehabilitation program |
| Cheema 2011 (19) | Not a prehabilitation program |
| Dijkstra-Eshuis 2015 (24) | Not a prehabilitation program |
| Dolin 2021 (25) | Not a prehabilitation program |
| Dunne 2014 (26) | Not a prehabilitation program |
| Dönmez 2017 (27) | Not a prehabilitation program |
| Fahim 2021 (28) | Not a prehabilitation program |
| Gillman 2022 (33) | Not a prehabilitation program |
| Gollhofer 2015 (34) | Not a prehabilitation program |
| Gravier 2022 (35) | Not a prehabilitation program |
| Heil 2021 (37) | Not a prehabilitation program |
| Institut Paoli-Calmettes 2020 (41) | Not a prehabilitation program |
| Jensen 2015 (42) | Not a prehabilitation program |
| Knight 2020 (50) | Not a prehabilitation program |
| Lai 2016 (51) | Not a prehabilitation program |
| Lee 2018 (56) | Not a prehabilitation program |
| Loh 2022 (59) | Not a prehabilitation program |
| Mader 2022 (61) | Not a prehabilitation program |
| Magno 2022 (62) | Not a prehabilitation program |
| McIsaac 2022 (65) | Not a prehabilitation program |
| Minnella 2020 (68) | Not a prehabilitation program |
| Ochalek 2018 (72) | Not a prehabilitation program |
| Paterson 2020 (77) | Not a prehabilitation program |
| Pavel 2020 (79) | Not a prehabilitation program |
| Sacomori 2021 (82) | Not a prehabilitation program |
| Schoentgen 2021 (84) | Not a prehabilitation program |
| Scriney 2022 (85) | Not a prehabilitation program |
| Sheill 2020 (88) | Not a prehabilitation program |
| Shi 2020 (89) | Not a prehabilitation program |
| Singh 2022 (91) | Not a prehabilitation program |
| Stefanelli 2013 (93) | Not a prehabilitation program |
| Steffens 2022 (94) | Not a prehabilitation program |
| St-Pierre 2022 (96) | Not a prehabilitation program |
| Sun 2020 (97) | Not a prehabilitation program |
| Tanaka 2017 (98) | Not a prehabilitation program |
| Valkenet 2017 (101) | Not a prehabilitation program |
| van Rooijen 2019 (102) | Not a prehabilitation program |
| Volz 2021 (103) | Not a prehabilitation program |
| Voorn 2021 (104) | Not a prehabilitation program |
| Waller 2020 (106) | Not a prehabilitation program |
| Wu 2019 (111) | Not a prehabilitation program |
| Yang 2012 (112) | Not a prehabilitation program |
| Özdemir 2019 (114) | Not a prehabilitation program |
| **Not a randomized study (n=20)** | |
| Baldoman 2018 (5) | Not a randomized study |
| Bordes 2015 (16) | Not a randomized study |
| Carli 2019 (18) | Not a randomized study |
| Cho 2008 (21) | Not a randomized study |
| Christensen 2019 (22) | Not a randomized study |
| Fatima 2022 (29) | Not a randomized study |
| Kaushik 2020 (46) | Not a randomized study |
| Kaye 2020 (47) | Not a randomized study |
| Kim 2014 (48) | Not a randomized study |
| Leong 2017 (57) | Not a randomized study |
| Loughney 2016 (60) | Not a randomized study |
| McCourt 2020 (64) | Not a randomized study |
| Milios 2019 (66) | Not a randomized study |
| Morano 2013 (69) | Not a randomized study |
| Shukla 2020 (90) | Not a randomized study |
| Tegels 2014 (99) | Not a randomized study |
| Vagvolgyi 2017 (100) | Not a randomized study |
| Wong 2017 (108) | Not a randomized study |
| Wright 2017 (109) | Not a randomized study |
| Zheng 2020 (113) | Not a randomized study |
| **No outcomes of interest (n=18)** | |
| Benzo 2011 (12) | No outcomes of interest |
| Bhatia 2019 (14) | No outcomes of interest |
| Blackwell 2020 (15) | No outcomes of interest |
| Demar-Wahnefried 2019 (23) | No outcomes of interest |
| Gillis 2018 (32) | No outcomes of interest |
| Hoeppner 2021 (38) | No outcomes of interest |
| Jones 2007 (44) | No outcomes of interest |
| Karenovics 2017 (45) | No outcomes of interest |
| Northgraves 2020 (71) | No outcomes of interest |
| Patel 2020 (75) | No outcomes of interest |
| Paterson 2021 (78) | No outcomes of interest |
| Postigo-Martin 2021 (80) | No outcomes of interest |
| Rydwik 2021 (81) | No outcomes of interest |
| Sebio 2022 (86) | No outcomes of interest |
| Sember 2017 (87) | No outcomes of interest |
| Wall 2000 (105) | No outcomes of interest |
| West 2021 (107) | No outcomes of interest |
| Wu 2021 (110) | No outcomes of interest |
| **Re-analysis of previous trial data=9** | |
| Awasthi 2018(4) | Re-analysis of previous trial data |
| Chen 2017 (20) | Re-analysis of previous trial data |
| Huang 2017 (40) | Re-analysis of previous trial data |
| Jensen 2016 (43) | Re-analysis of previous trial data |
| Klassen 2017 (49) | Re-analysis of previous trial data |
| Lai 2016b (52) | Re-analysis of previous trial data |
| Lai 2019 (54) | Re-analysis of previous trial data |
| Mayo 2011 (63) | Re-analysis of previous trial data |
| Minnella 2016 (67) | Re-analysis of previous trial data |
| **Article not available (n=5)** | |
| Flannigan 2020 (30) | Article not available |
| Galvão 2018 (31) | Article not available |
| \|Hiramatsu 2020 (36) | Article not available |
| Pameijer 2022 (74) | Article not available |
| Patel 2021 (76) | Article not available |
| **Intervention: radiotherapy + exercise (n=3)** | |
| Høgdal 2015 (39) | Intervention: radiotherapy + exercise |
| Schmidt 2015 (83) | Intervention: radiotherapy + exercise |
| Steindorf 2014 (95) | Intervention: radiotherapy + exercise |
| **Study protocol (n=2)** | |
| Lambaudie 2021 (55) | Study protocol |
| Sommer 2014 (92) | Study protocol |
| **Duplicate, companion (n=2)** | |
| Banerjee 2017 (6) | Duplicate, companion |
| Lai 2017 (53) | Duplicate, companion |
| **Intervention: chemotherapy + exercise (n=1)** | |
| Lin 2014 (58) | Intervention: chemotherapy + exercise |
| **Intervention: nutritional therapy + exercise (n=1)** | |
| Nakajima 2018 (70) | Intervention: nutritional therapy + exercise |
| **Not cancer (n=1)** | |
| Barberan 2017 (8) | Not cancer |

**References**

1. Adams SC, Herman J, Lega IC, Mitchell L, Hodgson D, Edelstein K, et al. Young Adult Cancer Survivorship: Recommendations for Patient Follow-up, Exercise Therapy, and Research. Vol. 5, JNCI Cancer Spectrum. 2021.

2. Allen S, Brown V, White D, King D, Hunt J, Prabhu P, et al. A randomised controlled trial of prehabilitation in patients receiving neoadjuvant therapy prior to resection for oesophagogastric (OG) cancer: effect on cardiorespiratory fitness, muscle mass and quality of life. British journal of surgery. 2020;107(SUPPL 4).

3. Ausania F, Senra P, Meléndez R, Caballeiro R, Ouviña R, Casal-Núñez E. Prehabilitation in patients undergoing pancreaticoduodenectomy: A randomized controlled trial. Revista Espanola de Enfermedades Digestivas. 2019;111(8).

4. Awasthi R, Minnella EM, Ferreira V, Ramanakumar A V., Scheede-Bergdahl C, Carli F. Supervised exercise training with multimodal pre-habilitation leads to earlier functional recovery following colorectal cancer resection. Acta Anaesthesiol Scand. 2019;63(4).

5. Baldoman D, Vandenbrink R. Physical Therapy Challenges in Head and Neck Cancer. In: Cancer Treatment and Research. 2018.

6. Banerjee S, Manley K, Shaw B, Lewis L, Cucato G, Mills R, et al. Vigorous intensity aerobic interval exercise in bladder cancer patients prior to radical cystectomy: a feasibility randomised controlled trial. Supportive Care in Cancer. 2018;26(5).

7. Barassi G, Bellomo RG, Di Iulio A, Lococo A, Porreca A, Di Felice PA, et al. Preoperative rehabilitation in lung cancer patients: Yoga approach. In: Advances in Experimental Medicine and Biology. 2018.

8. Barberan-Garcia A, Ubré M, Roca J, Lacy AM, Burgos F, Risco R, et al. Personalised Prehabilitation in High-risk Patients Undergoing Elective Major Abdominal Surgery : A Randomized Blinded Controlled Trial. Ann Surg. 2018;267(1).

9. Barberan-Garcia A, Navarro-Ripoll R, Sánchez-Lorente D, Moisés-Lafuente J, Boada M, Messaggi-Sartor M, et al. Cost-effectiveness of a technology-supported multimodal prehabilitation program in moderate-to-high risk patients undergoing lung cancer resection: Randomized controlled trial protocol. BMC Health Serv Res. 2020;20(1).

10. Baré M, Mora L, Pera M, Collera P, Redondo M, Escobar A, et al. Type and Consequences of Short-Term Complications in Colon Cancer Surgery, Focusing on the Oldest Old. Clin Colorectal Cancer. 2020;19(1).

11. Bausys A, Ümarik T, Luksta M, Reinsoo A, Rackauskas R, Anglickiene G, et al. Impact of the Interval Between Neoadjuvant Chemotherapy and Gastrectomy on Short- and Long-Term Outcomes for Patients with Advanced Gastric Cancer. Ann Surg Oncol. 2021;28(8).

12. Benzo R, Wigle D, Novotny P, Wetzstein M, Nichols F, Shen RK, et al. Preoperative pulmonary rehabilitation before lung cancer resection: Results from two randomized studies. Lung Cancer. 2011;74(3).

13. Berger-Richardson D, Alavi N, Trudeau M, Lemon-Wong S, Look-Hong N, Mascarenhas J, et al. A randomized controlled feasibility study comparing a multimodal prehabilitation protocol to normal care for women undergoing neo-adjuvant chemotherapy for breast cancer. Ann Surg Oncol. 2020;27 CC-.

14. Bhatia C, Kayser B. Preoperative high-intensity interval training is effective and safe in deconditioned patients with lung cancer: A randomized clinical trial. J Rehabil Med. 2019;51(9).

15. Blackwell JEM, Doleman B, Boereboom CL, Morton A, Williams S, Atherton P, et al. High-intensity interval training produces a significant improvement in fitness in less than 31 days before surgery for urological cancer: a randomised control trial. Prostate Cancer Prostatic Dis. 2020;23(4).

16. Bordes J, Cardinal M, Kaiser E. Prehabilitation versus Rehabilitation. Vol. 122, Anesthesiology. 2015.

17. Burgio KL, Goode PS, Urban DA, Umlauf MG, Locher JL, Bueschen A, et al. Preoperative biofeedback assisted behavioral training to decrease post-prostatectomy incontinence: A randomized, controlled trial. Journal of Urology. 2006;175(1).

18. Carli F, Feldman LS. From preoperative risk assessment and prediction to risk attenuation: a case for prehabilitation. Vol. 122, British Journal of Anaesthesia. 2019.

19. Cheema FN, Abraham NS, Berger DH, Albo D, Taffet GE, Naik AD. Novel approaches to perioperative assessment and intervention may improve long-term outcomes after colorectal cancer resection in older adults. Vol. 253, Annals of Surgery. 2011.

20. Chen BP, Awasthi R, Sweet SN, Minnella EM, Bergdahl A, Santa Mina D, et al. Four-week prehabilitation program is sufficient to modify exercise behaviors and improve preoperative functional walking capacity in patients with colorectal cancer. Supportive Care in Cancer. 2017;25(1).

21. Cho H, Tsuburaya A, Sakamoto J, Morita S, Oba K, Yoshikawa T, et al. A randomized phase II trial of preoperative exercise to reduce operative risk in gastric cancer patients with metabolic syndrome: Adjuvant exercise for general elective surgery (AEGES) study group. Jpn J Clin Oncol. 2008;38(1).

22. Christensen JF, Simonsen C, Banck-Petersen A, Thorsen-Streit S, Herrstedt A, Djurhuus SS, et al. Safety and feasibility of preoperative exercise training during neoadjuvant treatment before surgery for adenocarcinoma of the gastro-oesophageal junction. BJS Open. 2019;3(1).

23. Demark-Wahnefried W, Rogers LQ, Gibson JT, Harada S, Frugé AD, Oster RA, et al. Randomized trial of weight loss in primary breast cancer: Impact on body composition, circulating biomarkers and tumor characteristics. Int J Cancer. 2020;146(10).

24. Dijkstra-Eshuis J, Van Den Bos TWL, Splinter R, Bevers RFM, Zonneveld WCG, Putter H, et al. Effect of preoperative pelvic floor muscle therapy with biofeedback versus standard care on stress urinary incontinence and quality of life in men undergoing laparoscopic radical prostatectomy: A randomised control trial. Neurourol Urodyn. 2015;34(2).

25. Dolin TG, Mikkelsen M, Jakobsen HL, Nordentoft T, Pedersen TS, Vinther A, et al. Geriatric assessment and intervention in older vulnerable patients undergoing surgery for colorectal cancer: a protocol for a randomised controlled trial (GEPOC trial). BMC Geriatr. 2021;21(1).

26. Dunne D, Jones R, Lythgoe D, Malik H, Poston GJ, Jack S, et al. 111. Prehabilitation before liver surgery. European Journal of Surgical Oncology (EJSO). 2014;40(11).

27. Dönmez AA, Kapucu S. The effectiveness of a clinical and home-based physical activity program and simple lymphatic drainage in the prevention of breast cancer-related lymphedema: A prospective randomized controlled study. European Journal of Oncology Nursing. 2017;31.

28. Fahim M, Dijksman LM, Derksen WJM, Bloemen JG, Biesma DH, Smits AB. Prospective multicentre study of a new bowel obstruction treatment in colorectal surgery: Reduced morbidity and mortality. European Journal of Surgical Oncology. 2021;47(9).

29. Fatima T, Shakoor A, Ilyas M, Safdar M, Majeed S. Effectiveness of preoperative stretchings on postoperative shoulder function in patients undergoing mastectomy. J Pak Med Assoc. 2022;72(4).

30. Flannigan* R, Locke J, Schulz G, Campbell K, Van Patten C, Goldenberg L, et al. MP67-16 GETTING ADVANTAGE FROM THE “TEACHABLE MOMENT” AT INITIAL DIAGNOSIS OF PROSTATE CANCER - RESULTS OF A RANDOMIZED CONTROLLED PHYSICAL EXERCISE PROGRAM. Journal of Urology. 2020;203(Supplement 4).

31. Galvão D. Exercise medicine across the cancer trajectory. J Sci Med Sport. 2018;21.

32. Gillis C, Fenton TR, Sajobi TT, Minnella EM, Awasthi R, Loiselle SÈ, et al. Trimodal prehabilitation for colorectal surgery attenuates post-surgical losses in lean body mass: A pooled analysis of randomized controlled trials. Clinical Nutrition. 2019;38(3).

33. Gillman A, Hayes M, Sheaf G, Walshe M, Reynolds J V., Regan J. Exercise-based dysphagia rehabilitation for adults with oesophageal cancer: a systematic review. BMC Cancer. 2022;22(1).

34. Gollhofer SM, Wiskemann J, Schmidt ME, Klassen O, Ulrich CM, Oelmann J, et al. Factors influencing participation in a randomized controlled resistance exercise intervention study in breast cancer patients during radiotherapy. BMC Cancer. 2015;15(1).

35. Gravier FE, Smondack P, Boujibar F, Prieur G, Medrinal C, Combret Y, et al. Prehabilitation sessions can be provided more frequently in a shortened regimen with similar or better efficacy in people with non-small cell lung cancer: a randomised trial. J Physiother. 2022;68(1).

36. Hiramatsu Y, Kawata S, Watanabe K, Honke J, Shirai Y, Haneda R, et al. Clinical study on the usefulness of preoperative short-term program for nutrition and exercise before esophagectomy. Clin Nutr ESPEN. 2020;40.

37. Heil TC, Melis RJF, Maas HAAM, van Munster BC, Olde Rikkert MGM, de Wilt JHW, et al. Technical efficiency evaluation of colorectal cancer care for older patients in Dutch hospitals. PLoS One. 2021;16(12 December).

38. Hoeppner J, Plum PS, Buhr H, Gockel I, Lorenz D, Ghadimi M, et al. Surgical treatment of esophageal cancer—Indicators for quality in diagnostics and treatment. Vol. 92, Chirurg. 2021.

39. Hogdal N, Juhl C, Aadahl M, Gluud C. Early preventive exercises versus usual care does not seem to reduce trismus in patients treated with radiotherapy for cancer in the oral cavity or oropharynx: A randomised clinical trial. Acta Oncol (Madr). 2015;54(1).

40. Huang J, Lai Y, Zhou X, Li S, Su J, Yang M, et al. Short-term high-intensity rehabilitation in radically treated lung cancer: A three-armed randomized controlled trial. J Thorac Dis. 2017;9(7).

41. Tyran M, Fau P, Mailleux H, Eustache P, Benkreira M, Salem N, et al. Start of activity with the MRIdian® system: The first 200 patients treated at the Institut Paoli-Calmettes. Bull Cancer. 2021;108(11).

42. Jensen BT, Petersen AK, Jensen JB, Laustsen S, Borre M. Efficacy of a multiprofessional rehabilitation programme in radical cystectomy pathways: A prospective randomized controlled trial. Scand J Urol. 2015;49(2).

43. Jensen BT, Laustsen S, Jensen JB, Borre M, Petersen AK. Exercise-based pre-habilitation is feasible and effective in radical cystectomy pathways—secondary results from a randomized controlled trial. Supportive Care in Cancer. 2016;24(8).

44. Jones LW, Peddle CJ, Eves ND, Haykowsky MJ, Courneya KS, Mackey JR, et al. Effects of presurgical exercise training on cardiorespiratory fitness among patients undergoing thoracic surgery for malignant lung lesions. Cancer. 2007;110(3).

45. Karenovics W, Licker M, Ellenberger C, Christodoulou M, Diaper J, Bhatia C, et al. Short-term preoperative exercise therapy does not improve long-term outcome after lung cancer surgery: A randomized controlled study. European Journal of Cardio-thoracic Surgery. 2017;52(1).

46. Kaushik D, Shah P, Pratap KA, Thompson IM, Svatek RS, Hernandez J, et al. Abstract LB-292: A randomized controlled trial of yoga in men with prostate cancer undergoing radical prostatectomy. Cancer Res. 2020;80(16_Supplement).

47. Kaye DR, Schafer C, Thelen-Perry S, Parker C, Iglay-Reger H, Daignault-Newton S, et al. The Feasibility and Impact of a Presurgical Exercise Intervention Program (Prehabilitation) for Patients Undergoing Cystectomy for Bladder Cancer. Urology. 2020;145.

48. Kim I, Lee H. Effects of a progressive walking program on physical activity, exercise tolerance, recovery, and post-operative complications in patients with a lung resection. J Korean Acad Nurs. 2014;44(4).

49. Klassen O, Schmidt ME, Ulrich CM, Schneeweiss A, Potthoff K, Steindorf K, et al. Muscle strength in breast cancer patients receiving different treatment regimes. J Cachexia Sarcopenia Muscle. 2017;8(2).

50. Knight HP, Fong ZV, Qian CL, Kaslow-Zieve E, Azoba CC, Ferrone CR, et al. Patient-reported outcomes (PROs) in older adults with gastrointestinal (GI) cancer undergoing surgery. Journal of Clinical Oncology. 2020;38(29_suppl).

51. Lai Y, Su J, Yang M, Zhou K, Che G. Impact and effect of preoperative short-term pulmonary rehabilitation training on lung cancer patients with mild to moderate chronic obstructive pulmonary disease: A randomized trial. Chinese Journal of Lung Cancer. 2016;19(11).

52. Lai X Bin, Ching SSY, Wong FKY. Nurse-led cancer care: A scope review of the past years (2003-2016). Vol. 4, International Journal of Nursing Sciences. 2017.

53. Lai Y, Huang J, Yang M, Su J, Liu J, Che G. Seven-day intensive preoperative rehabilitation for elderly patients with lung cancer: a randomized controlled trial. Journal of Surgical Research. 2017;209.

54. Lai Y, Wang X, Zhou K, Su J, Che G. Impact of one-week preoperative physical training on clinical outcomes of surgical lung cancer patients with limited lung function: a randomized trial. Ann Transl Med. 2019;7(20).

55. Lambaudie E, Bannier/braticevic C, Villaron/goetgheluck C, Zemmour C, Boher JM, Ben Soussan P, et al. TRAINING-Ovary 01 (connecTed pRehabiliAtIoN pelvIc caNcer surGery): Multicenter randomized study comparing neoadjuvant chemotherapy for patients managed for ovarian cancer with or without a connected pre-habilitation program. International Journal of Gynecological Cancer. 2021;31(6).

56. Lee R, Yeo ST, Rogers SN, Caress AL, Molassiotis A, Ryder D, et al. Randomised feasibility study to compare the use of Therabite® with wooden spatulas to relieve and prevent trismus in patients with cancer of the head and neck. British Journal of Oral and Maxillofacial Surgery. 2018;56(4).

57. Leong KJ, Chapman MAS. Current data about the benefit of prehabilitation for colorectal cancer patients undergoing surgery are not sufficient to alter the NHS cancer waiting targets. Vol. 19, Colorectal Disease. 2017.

58. Lin KY, Shun SC, Lai YH, Liang JT, Tsauo JY. Comparison of the Effects of a Supervised Exercise Program and Usual Care in Patients with Colorectal Cancer Undergoing Chemotherapy. Cancer Nurs. 2014;37(2).

59. Loh EW, Shih HF, Lin CK, Huang TW. Effect of progressive muscle relaxation on postoperative pain, fatigue, and vital signs in patients with head and neck cancers: A randomized controlled trial. Patient Educ Couns. 2022;105(7).

60. Loughney L, West MA, Kemp GJ, Rossiter HB, Burke SM, Cox T, et al. The effects of neoadjuvant chemoradiotherapy and an in-hospital exercise training programme on physical fitness and quality of life in locally advanced rectal cancer patients (The EMPOWER Trial): Study protocol for a randomised controlled trial. Trials. 2016;17(1).

61. Mader T, Chaillou T, Alves ES, Jude B, Cheng AJ, Kenne E, et al. Exercise reduces intramuscular stress and counteracts muscle weakness in mice with breast cancer. J Cachexia Sarcopenia Muscle. 2022;13(2).

62. Magno S, Rossi MM, Filippone A, Rossi C, Guarino D, Maggiore C, et al. Screening for Physical Activity Levels in Non-Metastatic Breast Cancer Patients Undergoing Surgery: An Observational Study. Integr Cancer Ther. 2022;21.

63. Mayo NE, Feldman L, Scott S, Zavorsky G, Kim DJ, Charlebois P, et al. Impact of preoperative change in physical function on postoperative recovery: Argument supporting prehabilitation for colorectal surgery. Surgery. 2011;150(3).

64. McCourt O, Fisher A, Ramdharry G, Roberts AL, Land J, Rabin N, et al. PERCEPT myeloma: A protocol for a pilot randomised controlled trial of exercise prehabilitation before and during autologous stem cell transplantation in patients with multiple myeloma. BMJ Open. 2020;10(1).

65. McIsaac DI, Hladkowicz E, Bryson GL, Forster AJ, Gagne S, Huang A, et al. Home-based prehabilitation with exercise to improve postoperative recovery for older adults with frailty having cancer surgery: the PREHAB randomised clinical trial. Br J Anaesth. 2022;129(1).

66. Milios JE, Ackland TR, Green DJ. Pelvic floor muscle training in radical prostatectomy: A randomized controlled trial of the impacts on pelvic floor muscle function and urinary incontinence. BMC Urol. 2019;19(1).

67. Minnella EM, Awasthi R, Gillis C, Fiore JF, Liberman AS, Charlebois P, et al. Patients with poor baseline walking capacity are most likely to improve their functional status with multimodal prehabilitation. Surgery (United States). 2016;160(4).

68. Minnella EM, Ferreira V, Awasthi R, Charlebois P, Stein B, Liberman AS, et al. Effect of two different pre-operative exercise training regimens before colorectal surgery on functional capacity: A randomised controlled trial. Eur J Anaesthesiol. 2020;37(11).

69. Morano MT, Araújo AS, Nascimento FB, Da Silva GF, Mesquita R, Pinto JS, et al. Preoperative pulmonary rehabilitation versus chest physical therapy in patients undergoing lung cancer resection: A pilot randomized controlled trial. Arch Phys Med Rehabil. 2013;94(1).

70. Nakajima H, Yokoyama Y, Inoue T, Nagaya M, Mizuno Y, Kadono I, et al. Clinical Benefit of Preoperative Exercise and Nutritional Therapy for Patients Undergoing Hepato-Pancreato-Biliary Surgeries for Malignancy. Ann Surg Oncol. 2019;26(1).

71. Northgraves MJ, Arunachalam L, Madden LA, Marshall P, Hartley JE, MacFie J, et al. Feasibility of a novel exercise prehabilitation programme in patients scheduled for elective colorectal surgery: a feasibility randomised controlled trial. Supportive Care in Cancer. 2020;28(7).

72. Ochalek K, Gradalski T, Szygula Z, Partsch H. Physical Activity with and Without Arm Sleeves: Compliance and Quality of Life after Breast Cancer Surgery-A Randomized Controlled Trial. Lymphat Res Biol. 2018;16(3).

73. Onerup A, Angenete E, Bock D, Börjesson M, Fagevik Olsén M, Grybäck Gillheimer E, et al. The effect of pre- and post-operative physical activity on recovery after colorectal cancer surgery (PHYSSURG-C): Study protocol for a randomised controlled trial. Trials. 2017;18(1).

74. Pameijer CR. Exercise Intervention Prior to CRS-HIPEC: Feasibility & Impact.

75. Patel VG, Oh WK, Galsky MD. Treatment of muscle‐invasive and advanced bladder cancer in 2020. CA Cancer J Clin. 2020;70(5).

76. Patel YS, Churchill IF, Sullivan KA, Beauchamp M, Wald J, Mbuagbaw L, et al. OA04.01 Move For Surgery – A Novel Preconditioning Program to Optimize Health Before Thoracic Surgery: A Randomized Controlled Trial. Journal of Thoracic Oncology. 2021;16(10).

77. Paterson C, Primeau C, Howard N, Xiberras P, Pillay B, Crowe H. Experiences of Unmet Need and Access to Supportive Care for Men Receiving Androgen Deprivation Therapy for Prostate Cancer: A Bi-national Study. Vol. 36, Seminars in Oncology Nursing. 2020.

78. Paterson C, Kozlovskaia M, Turner M, Strickland K, Roberts C, Ogilvie R, et al. Identifying the supportive care needs of men and women affected by chemotherapy-induced alopecia? A systematic review. Vol. 15, Journal of Cancer Survivorship. 2021.

79. Pavel MC, Casanova R, Estalella L, Memba R, Llàcer-Millán E, Achalandabaso M, et al. The effect of preoperative chemotherapy on liver regeneration after portal vein embolization/ligation or liver resection in patients with colorectal liver metastasis: a systematic review protocol. Syst Rev. 2020;9(1).

80. Postigo-Martin P, Peñafiel-Burkhardt R, Gallart-Aragón T, Alcaide-Lucena M, Artacho-Cordón F, Galiano-Castillo N, et al. Attenuating treatment-related cardiotoxicity in women recently diagnosed with breast cancer via a tailored therapeutic exercise program: Protocol of the atope trial. Phys Ther. 2021;101(3).

81. Rydwik E, Anmyr L, Regardt M, McAllister A, Zarenoe R, Åkerman E, et al. ReCOV: recovery and rehabilitation during and after COVID-19 – a study protocol of a longitudinal observational study on patients, next of kin and health care staff. BMC Sports Sci Med Rehabil. 2021;13(1).

82. Sacomori C, Lorca LA, Martinez-Mardones M, Salas-Ocaranza RI, Reyes-Reyes GP, Pizarro-Hinojosa MN, et al. A randomized clinical trial to assess the effectiveness of pre- and post-surgical pelvic floor physiotherapy for bowel symptoms, pelvic floor function, and quality of life of patients with rectal cancer: CARRET protocol. Trials. 2021;22(1).

83. Schmidt ME, Meynköhn A, Habermann N, Wiskemann J, Oelmann J, Hof H, et al. Resistance Exercise and Inflammation in Breast Cancer Patients Undergoing Adjuvant Radiation Therapy: Mediation Analysis from a Randomized, Controlled Intervention Trial. Int J Radiat Oncol Biol Phys. 2016;94(2).

84. Schoentgen N, Califano G, Manfredi C, Romero-Otero J, Chun FKH, Ouzaid I, et al. Is it Worth Starting Sexual Rehabilitation Before Radical Prostatectomy? Results From a Systematic Review of the Literature. Vol. 8, Frontiers in Surgery. 2021.

85. Scriney A, Russell A, Loughney L, Gallagher P, Boran L. The impact of prehabilitation interventions on affective and functional outcomes for young to midlife adult cancer patients: A systematic review. Vol. 31, Psycho-Oncology. 2022.

86. Sebio Garcia R. Prehabilitation Program Based on Health Education and Nordic Walking to Reduce Musculoskeletal Impairments in Women Undergoing Breast Cancer Surgery. 2022.

87. Sember A, Pranskevich C, Scott ST, Hutchinson I V., Hoffman R. Prehabilitation for Lymphedema in head and neck cancer patients at a community cancer center. Journal of Community and Supportive Oncology. 2017;15(3).

88. Sheill G, Guinan E, O’Neill L, Normand C, Doyle SL, Moore S, et al. Preoperative exercise to improve fitness in patients undergoing complex surgery for cancer of the lung or oesophagus (PRE-HIIT): Protocol for a randomized controlled trial. BMC Cancer. 2020;20(1).

89. Shi Q, Diao Y, Qian J. Application of single-hole thoracoscopic surgery combined with eras concept for respiratory function exercise in perioperative period of lung cancer. Chinese Journal of Lung Cancer. 2020;23(8).

90. Shukla A, Granger C, Edbrook L, Wright G, Denehy L. Prehabilitation for Individuals Having Lung Cancer Surgery: Feasibility and Acceptability of A Pre-Operative Exercise Intervention. Respirology. 2020;25.

91. Singh B, Toohey K. The effect of exercise for improving bone health in cancer survivors — A systematic review and meta-analysis. Vol. 25, Journal of Science and Medicine in Sport. 2022.

92. Sommer MS, Trier K, Vibe-Petersen J, Missel M, Christensen M, Larsen KR, et al. Perioperative rehabilitation in operation for lung cancer (PROLUCA) - rationale and design. BMC Cancer. 2014;14(1).

93. Stefanelli F, Meoli I, Cobuccio R, Curcio C, Amore D, Casazza D, et al. High-intensity training and cardiopulmonary exercise testing in patients with chronic obstructive pulmonary disease and non-small-cell lung cancer undergoing lobectomy. European Journal of Cardio-thoracic Surgery. 2013;44(4).

94. Steffens D, Young J, Riedel B, Morton R, Denehy L, Heriot A, et al. PRehabIlitatiOn with pReoperatIve exercise and educaTion for patients undergoing major abdominal cancer surgerY: protocol for a multicentre randomised controlled TRIAL (PRIORITY TRIAL). BMC Cancer. 2022;22(1).

95. Steindorf K, Schmidt ME, Klassen O, Ulrich CM, Oelmann J, Habermann N, et al. Randomized, controlled trial of resistance training in breast cancer patients receiving adjuvant radiotherapy: Results on cancer-related fatigue and quality of life. Annals of Oncology. 2014;25(11).

96. St-Pierre J, Drummond K, Minella E, Scheede-Bergdahl C, Ferri L, Carli F. Feasibility of multimdodal prehabilitation to enhance preoperative functional capacity of esophageal cancer patients during concurrent neoadjuvant chemotherapies - a pilot interventional study. European Journal of Surgical Oncology. 2022;48(2).

97. Sun Y, Pang YH, Mao NQ, Luo JN, Cai DL, Chen FF. [Effect of transcutaneous electrical acupoint stimulation on venous thrombosis after lung cancer surgery: a randomized controlled trial]. Zhongguo Zhen Jiu. 2020;40(12).

98. Tanaka R, Lee SW, Kawai M, Tashiro K, Kawashima S, Kagota S, et al. Protocol for enhanced recovery after surgery improves short-term outcomes for patients with gastric cancer: a randomized clinical trial. Gastric Cancer. 2017;20(5).

99. Tegels JJW, De Maat MFG, Hulsewé KWE, Hoofwijk AGM, Stoot JH. Improving the outcomes in gastric cancer surgery. Vol. 20, World Journal of Gastroenterology. 2014.

100. Vagvolgyi A, Rozgonyi Z, Kerti M, Vadasz P, Varga J. Effectiveness of perioperative pulmonary rehabilitation in thoracic surgery. J Thorac Dis. 2017;9(6).

101. Valkenet K, Trappenburg JCA, Ruurda JP, Guinan EM, Reynolds J V., Nafteux P, et al. Multicentre randomized clinical trial of inspiratory muscle training versus usual care before surgery for oesophageal cancer. British Journal of Surgery. 2018;105(5).

102. Van Rooijen S, Carli F, Dalton S, Thomas G, Bojesen R, Le Guen M, et al. Multimodal prehabilitation in colorectal cancer patients to improve functional capacity and reduce postoperative complications: The first international randomized controlled trial for multimodal prehabilitation. BMC Cancer. 2019;19(1).

103. Volz S, Koch F, Dayan D, Upadhyay M, Otto S, Schochter F, et al. Is there evidence behind pre- or perioperative cognitive training in gynaecological patients on the prevention of perioperative cognitive dysfunction? A review. Archives of Gynecology and Obstetrics. 2021.

104. Voorn MJJ, Beukers K, Trepels CMM, Bootsma GP, Bongers BC, Janssen-Heijnen MLG. Associations between pretreatment nutritional assessments and treatment complications in patients with stage I-III non-small cell lung cancer: A systematic review. Clin Nutr ESPEN. 2022;47.

105. Wall LM. Changes in Hope and Power in Lung Cancer Patients Who Exercise. Nurs Sci Q. 2000;13(3).

106. Waller E, Rahman S, Sutton P, Allen J, Saxton J, Aziz O. Randomised controlled trial of patients undergoing prehabilitation with wearables versus standard of care before major abdominal cancer surgery (Trial Registration: NCT04047524). Colorectal disease. 2020;22(SUPPL 1).

107. West MA, Jack S, Grocott MPW. Prehabilitation before surgery: Is it for all patients? Vol. 35, Best Practice and Research: Clinical Anaesthesiology. 2021.

108. Wong SG, Maida E, Harvey D, Wagner N, Sonnadara R, Amin N. Evaluation of a physiatrist-directed prehabilitation intervention in frail patients with colorectal cancer: A randomised pilot study protocol. BMJ Open. 2017;7(6).

109. Wright EJ. Exercising patient-centredness in prehabilitation programs. Vol. 43, European Journal of Surgical Oncology. 2017.

110. Fiona W, Oloruntobi R, Roberto LC, Tarannum R. The feasibility and effects of a telehealth-delivered home-based prehabilitation program for cancer patients during the pandemic. Current Oncology. 2021;28(3).

111. Wu XD, Fu CF, Chen YL, Kong LH, Pan ZZ, Zheng MC. Intervention effect of biofeedback combined with pelvic floor muscle exercise on low anterior resection syndrome in patients with low anus-preserving rectal cancer. Zhonghua Yi Xue Za Zhi. 2019;99(30).

112. Yang EJ, Lim JY, Rah UW, Kim YB. Effect of a pelvic floor muscle training program on gynecologic cancer survivors with pelvic floor dysfunction: A randomized controlled trial. In: Gynecologic Oncology. 2012.

113. Zheng Y, Mao M, Ji M, Zheng Q, Liu L, Zhao Z, et al. Does a pulmonary rehabilitation based ERAS program (PREP) affect pulmonary complication incidence, pulmonary function and quality of life after lung cancer surgery? Study protocol for a multicenter randomized controlled trial. BMC Pulm Med. 2020;20(1).

114. Özdemir IA, Comba C, Demirayak G, Gülseren V, Erdogan SV, Aslanova F, et al. Impact of pre-operative walking on post-operative bowel function in patients with gynecologic cancer. International Journal of Gynecological Cancer. 2019;29(8).
