# Supplementary material for: Prehabilitation programs for individuals with cancer: a systematic review of randomized-controlled trials
Source: Syst Rev. 2023 Nov 17;12:219. doi: 10.1186/s13643-023-02373-4 (PMC10655304; doi:10.1186/s13643-023-02373-4)
Supplement: Supplementary file 4 — Additional file 4. Characteristics of the ongoing studies. [file 13643_2023_2373_MOESM4_ESM.docx]

**Additional file 4. Characteristics of ongoing studies**

# **Dalton 2020**

| **Trial name or title** | Neo-Train: Pre-operative Exercise During Neoadjuvant Chemotherapy in Patients with Breast Cancer - a Randomized Controlled Trial |
| --- | --- |
| **Objective** | To investigate the effects of supervised pre-operative aerobic and resistance exercise in patients with breast cancer during neoadjuvant chemotherapy. |
| **Methods** | Design: Parallel Assignment (2 groups) |
| **Participants** | Inclusion:   - Patients newly diagnosed with histologically verified breast cancer and scheduled for neoadjuvant chemotherapy. - Female gender - Aged ≥ 18 years old. - Signed informed consent.   Exclusion:   - Patients ineligible for or who have declined to receive neoadjuvant chemotherapy - Contraindications to magnetic resonance imaging (MRI) - Physical or cognitive disabilities preventing exercise or physical testing. - Inability to read and understand Danish. - Based on clinical judgement, the physician assesses that the patient is not suitable for inclusion |
| **Interventions** | Group 1: Behavioral prehabilitation program  Group 2: Usual care and a weekly phone call for attention control  Estimated Enrollment: 45 participants in total |
| **Outcomes** | Tumour size and change in the maximum diameter (MRI) (Primary outcome)  Secondary outcomes  Relative dose intensity of neoadjuvant chemotherapy (percentage)  Number of participants with neoadjuvant chemotherapy dose reductions (percentage)  Number of participants with neoadjuvant chemotherapy dose delays (percentage)  Number of participants with early discontinuation of neoadjuvant chemotherapy (percentage)  Number of hospital admissions during neoadjuvant chemotherapy (percentage)  Total length of hospital admissions during neoadjuvant chemotherapy (days)  Total body mass (bioelectrical impedance)  Lean body mass (bioelectrical impedance)  Fat mass (bioelectrical impedance)  Physical fitness (progressive cycle ergometer test - watt max test)  Muscle strength (1 RM on leg press and pull down)  Physical function (hand-held dynamometer)  Level of physical activity (wearable objective measurement device)  Health-related quality of life (the Functional Assessment of Cancer Therapy - Breast Cancer (FACT-B))  General anxiety (Generalised Anxiety Disorder 7-item (GAD-7)).  Depression (the Patient Health Questionnaire-9 (PHQ-9))  Psychological distress (the Distress Thermometer (DT))  Post-operative referral to and participation in municipal rehabilitation programs (adherence)  Tumour size (clinical examination of the breast)  Tumour size (Measured by a pathologist from the tumour surgical specimen at breast surgery)  Pathological response grade (Measured by a pathologist from the tumour surgical specimen at breast surgery)  Tumour infiltrating lymphocyte population (Measured by a pathologist from the tumour surgical specimen at breast surgery)  Tumour vascularity (Measured by a pathologist from the tumour surgical specimen at breast surgery)  Liquid biopsies (blood samples)  Metabolic and inflammatory markers (blood samples)  Cytokines (blood samples)  Cell proliferation (blood samples) |
| **Starting date** | Start date: January 2020  Completion date: December 2023 |
| **Contact information** | Correspondence: Susanne Dalton, Prof. Eva Kjeldsted, MSc  Tel: +45 30381540  E-mail: [sdalt@regionsjaelland.dk](mailto:sdalt@regionsjaelland.dk); [evakj@regionsjaelland.dk](mailto:evakj@regionsjaelland.dk)  Department of Clinical Oncology and Palliative Care, Zealand University Hospital. Naestved, Denmark |
| **Notes** | Status: Recruiting  The trial was registered prospectively at ClinicalTrials.gov (NCT04623554) on November 10, 2020.  <https://clinicaltrials.gov/ct2/show/study/NCT04623554>. |

# **Palma 2020**

| **Trial name or title** | Feasibility and Acceptance of High Intensity Interval Training in the Prehabilitation of Patients Suffering From Gynecological Cancers - a Randomized Controlled Pilot Study |
| --- | --- |
| **Objective** | to investigate the feasibility and acceptance of a prehabilitation training intervention with high-intensity interval training compared to a conventional moderate intensity continuous training and a non-training collective. Secondary goals are the investigation of the effectiveness of threshold-based intensity prescriptions and the impact on quality of life, fatigue, anxiety, depression, sexuality and ability to work. |
| **Methods** | Design: Parallel Assignment (2 groups) |
| **Participants** | Inclusion:   - histopathologically verified cervical carcinoma (ICD10-C53) or - endometrial carcinoma (ICD10-C54) or - ovarian carcinoma (ICD10-C56) - planned primary/adjuvant surgery or medical training therapy - oncological release for medical training therapy - Eastern cooperative oncology group performance status (ECOG) 0-II - Age >18a to 80 - mental aptitude to participate in the study.   Exclusion:   - serious orthopaedic, rheumatological, neurological, oncological and cardiovascular diseases which are incompatible with a training intervention - general contraindications for exercise ergometry according to the guidelines of the austrian society of cardiology - osseous or cerebral metastasis - participation in a training study in the last year or already before cancer diagnosis with high training volumes (>150min moderate / >75min more intensive endurance training per week) |
| **Interventions** | Group 1: Prehabilitation using bicycle ergometer training with high intensity interval group.  Group 2: Prehabilitation using bicycle ergometer training with moderate continuous intensity group.  Estimated Enrollment: 45 participants in total |
| **Outcomes** | adherence to the exercise intervention (rate of completed exercise sessions) (Primary outcome)  Secondary outcomes  Cardiovascular fitness (VO2max)  Workability (workability index questionnaire)  Health related Quality of life (EORTC-30)  Anxiety and depression (Hospital Anxiety and Depression Scale questionnaire)  Handgrip strength (dynamometer)  Body composition (bioimpedance analysis)  Cholesterol (Laboratory parameter)  Thyreoglobulin (Laboratory parameter  Hemoglobin A1c (Laboratory parameter)  Brain natriuretic peptide (Laboratory parameter)  Troponin T (Laboratory parameter)  Creatin Kinase (Laboratory parameter)  C-reactive protein (Laboratory parameter)  Interleukin 1 (Laboratory parameter)  Interleukin 6 (Laboratory parameter)  Tumor Necrosis Factor alpha (Laboratory parameter) |
| **Starting date** | Start date: January 2021  Completion date: January 2023 |
| **Contact information** | Correspondence: Stefano Palma, MD.  Tel: 434040023080  E-mail: stefano.palma@meduniwien.ac.at  Medical University of Vienna, Waehringer Guertel 18-20, 1090 Vienna, Austria |
| **Notes** | Status: Unknown  The trial was registered prospectively at ClinicalTrials.gov (NCT04618094) on November 5, 2020.  <https://clinicaltrials.gov/ct2/show/NCT04618094> |

# **Fernández- Bobadilla 2020**

| **Trial name or title** | Supervised Exercise for Head and Neck Cancer Patients Initiated Previously or After Treatment: the SEHNeCa Randomized Controlled Trial |
| --- | --- |
| **Objective** | To evaluate the effectiveness and efficiency of an innovative supervised exercise program for patients with head and neck cancer (SEHNeCa) to ameliorate loss of lean body mass, functional capacity and quality of life during one year, compared to a reference group receiving a physical activity prescription to be performed autonomously.  To identify the optimal timing for applying the supervised exercise program: in a Prehabilitation period, at least 2 weeks before starting the conventional chemoradiotherapy treatment and concomitant with it, or during a Rehabilitation period, starting 12 weeks after the first radiotherapy session, once standard treatment has finished. |
| **Methods** | Design: multicenter, parallel assignment (3 groups) |
| **Participants** | Inclusion:  Histological diagnosed locally advanced stage III-IVa-b with squamous cell carcinomas of the larynx, pharynx, oral cavity, salivary or in neck lymph nodes from an unknown primary tumour (TNM 9th AJCC classification; 2019.) treated with curative intent undergoing radiotherapy with or without concomitant chemotherapy (with or without previous surgery).   - Age: more than 18 years - WHO performance status of 0-1 (IK 80%) - Body mass index: more than 18.5. - No evidence of metastatic disease - No excessive alcohol intake (men > 21 and women > 14 units/week) - No current or previous malignancies that could prevent participation and training - No recent systematic resistance training.   Exclusion:   - Decompensated heart disease, uncontrolled hypertension (TAS>200 o TAD>110), cardiac insufficiency (NYHA II o mayor), heart failure or constrictive pericarditis, Neutropenia, severe anemia ( Hb<8.0 g/dl), platelets count <50.000 microL - Other health problems in which exercise is contraindicated. - Carry out physical activity regularly (150 min/week of moderate activity or 75 of vigorous activity), measured with the PVS questionarie. - Pregnancy - Tracheostomy. |
| **Interventions** | Group 1: Supervised exercise program group (before starting the conventional chemoradiotherapy)  Group 2: Supervised exercise program group (after the first radiotherapy session)  Group 3: Exercise prescription performed autonomously  Estimated Enrollment: 120 participants in total |
| **Outcomes** | Change in body lean mass (multi-frequency impedance) (Primary outcome)  Secondary outcomes  Quality of life (EORTCQLQ-C-30, QLQ-C-30, SF-36)  Functional capacity (six-minute walking test, pain disability index, FACIT-F test )  Strength (manual dynamometer)  Anxiety and depression (Hospital Anxiety and Depression Scale questionnaire)  Handgrip strength (dynamometer) |
| **Starting date** | Start date: December 2020  Completion date: September, 2021 |
| **Contact information** | Correspondence: Jon Cacicedo Fernández Bobadilla  Tel: NR  E-mail: NR  Biocruces Bizkaia research health institute, Barakaldo, Bizkaia, Spain |
| **Notes** | Status: Active, not recruiting  The trial was registered prospectively at ClinicalTrials.gov (NCT04658706) on December 8, 2020.  <https://clinicaltrials.gov/ct2/show/NCT04658706> |

# **Behrens 2020**

| **Trial name or title** | Resistance training as prehabilitation exercise in Mamma-CA |
| --- | --- |
| **Objective** | To test whether pre-rehabilitation hypertrophy training consisting of strength training for the upper extremities (m. Biceps brachii, m. Triceps brachii, m. Pectoralis) in combination with vibration training for hands and feet has a positive effect on the postoperative recovery process (End point: post-op complications). |
| **Methods** | Design: Parallel Assignment (2 groups) |
| **Participants** | Inclusion:   - diagnosed breast cancer - at least 12 weeks prior to surgical therapy (breast-conserving therapy (BET) or radical mastectomy with axillary dissection) - neo-adjuvant chemotherapy or neo-adjuvant radio-chemotherapy - Written and valid declaration of consent from the patient - Age> = 18 years   Exclusion:   - Neo-adjuvant radiotherapy alone - Relapse - Second malignancies - WHO / ECOG performance status> 1 - Life expectancy <5 months - Ductal carcinoma - Metastases - All illness situations that do not allow hypertrophy training, in particular: - Clinically manifest heart failure (NYHA III-IV) - Partial or global respiratory failure - Permanent thrombocytopenia <10,000 / µl, e.g. refractory autoimmune thrombocytopenias. - Congenital or acquired thrombocytopathies or coagulation disorders. - Symptomatic CHD (exercise ECG recommended if necessary) - Severe refractory hypertension - Non-adjustable COPD - Severe osteoporosis - Acute migraines, epilepsy, dizziness - Taking antidepressants (SSRIs) and other drugs that affect quality of life - Medical or psychological condition (psychomotor deficits) that does not allow the patient to take part in the study - Unwillingness to pass on personal illness data within the framework of the protocol |
| **Interventions** | Group 1: Resistance-hypertrophie exercise group  Group 2: homeworkout and relaxation exercise group  Estimated Enrollment: 64 participants in total |
| **Outcomes** | Post-operative complications (Primary outcome)  Secondary outcomes  quality of life (EORTC QLQ-C30, BR23)  fatigue syndrome (MFI 20)  Reduction of pain, sleep disorders, anxiety, depression, nausea and vomiting (HADS Score)  Reduction of postmenopausal symptoms  Influence on lymphedema and shoulder-arm morbidity  Improvement of the strength level (hand grip strength test)  Number of days in hospital  Influence on chemotherapy-induced polyneuropathies PNP (FACT CogNXT)  Movement behavior (BSA) |
| **Starting date** | Start date: December 2020  Completion date: NR |
| **Contact information** | Correspondence: Freerk Baumann; Joshua Behrens  Tel: +49221/478-32993; +49221/478-32993  E-mail: [freerk.baumann@uk-koeln.de](mailto:freerk.baumann@uk-koeln.de); joshua.behrens@uk-koeln.de  Uniklinik Köln, Centrum für integrierte Onkologie (CIO), Kerpener Str. 62 50937 Köln Germany |
| **Notes** | Status: Recruiting  The trial was registered prospectively at ICTRP (DRKS00023243) on December 23, 2020.  <https://trialsearch.who.int/Trial2.aspx?TrialID=DRKS00023243> |

# **Pameijer 2021**

| **Trial name or title** | Exercise Intervention Prior to CRS-HIPEC: Feasibility & Impact |
| --- | --- |
| **Objective** | To initiate an exercise program for patients with advanced GI or Gyn cancer, as prehabilitation prior to surgery. |
| **Methods** | Design: Parallel Assignment (2 groups) |
| **Participants** | Inclusion:   - Patients with peritoneal metastasis from appendix, colon, small bowel, endometrium, ovary or any other GI or Gyn primary tumor - Patients should be eligible for and consent to surgery with the intention of performing CRS / HIPEC at Penn State Hershey Medical Center. Eligibility for CRS/HIPEC is a clinical determination based on a combination of factors including radiographic extent of disease, tumor histology and medical fitness for surgery - Subjects who are explored but found to be unresectable will be included. - Male or female. - Patients should be age 18 or above. - Surgery is planned for not less than 6 weeks and no more than 10 weeks from consultation   Exclusion:   - Patients who do not consent to surgery. - Patients who require urgent surgery, sooner than 6 weeks - Patients receiving neoadjuvant chemotherapy, with no surgical date planned before 10 weeks from consultation. These patients can be enrolled at the time of the follow-up visit, as long as they meet above criteria - Patients not fluent in English - Pregnant women - Prisoners - Patients with Cognitive Impairment |
| **Interventions** | Group 1: Home-based exercise program prior to CRS-HIPEC exercise group  Group 2: Exercise education  Estimated Enrollment: 30 participants in total |
| **Outcomes** | Acceptability (Rate of enrollment into this trial) and Feasibility (The percent of exercises completed by subjects) (Primary outcome)  Secondary outcomes  30-day Complication (NR)  Quality of Life (NR) |
| **Starting date** | Start date: February 2021  Completion date: February 2024 |
| **Contact information** | Correspondence: Colette R Pameijer, MD; Jorge Benavides, MPH  Tel: 717-531-5272; 717-531-0003 ext 289441  E-mail: [cpameijer@pennstatehealth.psu.edu](mailto:cpameijer@pennstatehealth.psu.edu); jub1647@psu.edu  Hershey, Pennsylvania, 17033, United States |
| **Notes** | Status: Recruiting  The trial was registered prospectively at ClinicalTrials.gov (NCT04731441) on February 1, 2021  <https://clinicaltrials.gov/ct2/show/NCT04731441> |

# **Lin 2021**

| **Trial name or title** | A Prehabilitation Program to Boost Postoperative Functional Capacity in Surgical Lung Cancer Patients: a Randomized Controlled Trial |
| --- | --- |
| **Objective** | To evaluating a prehabiliation intervention designed to improve postoperative functional capacity (measured by 6-minure walking distance) in individuals undergoing lung resection for cancer. |
| **Methods** | Design: Parallel Assignment (2 groups) |
| **Participants** | Inclusion:   - Patients who are diagnosed of stage I, II, or IIIA Non-small-cell lung carcinoma (NSCLC) diagnosis, with or without pre-operative histologic confirmation - Patients scheduled to undergo lung resection surgery at least two weeks from recruitment - Patients with a Eastern Cooperative Oncology Group (ECOG) score of 0 or 1 at recruitment - Patients with no evidence of recurrent or progressive disease - Patients aged 18 or above - Patients able to communicate in Cantonese, Mandarin, or English - Patients is absence of any cognitive impairment - Patients with a score of 6 minutes walking test (6MWT) ≤ 500 meters at baseline. [rationale: a threshold value of 500 meters preoperative 6MWT predicts a higher risk of postoperative complications and prolonged LOS after lung resection]   Exclusion:   - Patients who had engaged in at least 150 minutes of moderate aerobic activity or 75 minutes of vigorous aerobic activity per week in the past three months - Presence of another concurrent, actively treated malignancy - Presence of chronic obstructive pulmonary disease - Presence of significant comorbidities that impede ability to engage in exercise, such as congestive heart failure, orthopedic disorders of the lower limbs, respiratory failure, or the need for portable oxygen therapy for activities of daily living |
| **Interventions** | Group 1: Supervised exercise plus unsupervised respiratory muscle training group  Group 2: Health education control  Estimated Enrollment: 198 participants in total |
| **Outcomes** | 6-minute walking distance (Primary outcome)  Secondary outcomes  Length of postoperative hospital stay (NR)  Postoperative complication (NR)  functional assessment of cancer treatment - Lung (FACT-L)  Change from baseline international physical activity questionnaire (IPAQ)  Daily step count (pedometer) |
| **Starting date** | Start date: April 2021  Completion date: May 2023 |
| **Contact information** | Correspondence: Chia-chin Lin, PhD; Xinyi Xu, BSN  Tel: 39176614; 39176948  E-mail: [cclin@hku.hk](mailto:cclin@hku.hk); [xuxinyi@connect.hku.hk](mailto:xuxinyi@connect.hku.hk)  Queen Mary Hospital, Hong Kong, Hong Kong |
| **Notes** | Status: Recruiting  The trial was registered prospectively at ClinicalTrials.gov (NCT04826835) on April 1, 2021  <https://clinicaltrials.gov/ct2/show/NCT04826835> |

# **Rydwik 2021**

| **Trial name or title** | Optimizing Physical Function Before Surgery: Effects on Complications and Physical Function After Gastrointestinal Cancer Surgery in Older People at Risk- A Randomized Controlled Trial |
| --- | --- |
| **Objective** | to explore the effects of preoperative exercise on physical fitness, postoperative complications, recovery, and health-related quality of life in older individuals at risk scheduled to undergo colorectal cancer surgery. |
| **Methods** | Design: Multicenter, parallel Assignment (2 groups) |
| **Participants** | Inclusion:   - Scheduled surgery due to colorectal cancer or liver metastases from colorectal cancer - Age ≥65 - A maximal walking speed below 2 meters per second - Understands and speaks the Swedish language   Exclusion:   - Planned hyperthermic intraperitoneal chemotherapy (HIPEC) procedure or flap surgery - Health conditions that prevent participation in assessment or exercise. Such conditions include, but are not limited to, unstable heart disease, severe systematic illness or orthopaedic conditions that may prohibit exercise. - The need for surgery within 2 weeks - Permanent wheelchair user - Residence outside of Stockholm County |
| **Interventions** | Group 1: Prehabilitation high-intensity, home-based, exercise program group  Group 2: Usual care  Estimated Enrollment: 120 participants in total |
| **Outcomes** | Postoperative Complications 30 days post-surgery (The Clavien-Dindo classification) and maximal walking distance (The 6-minute walk test) (Primary outcome)  Secondary outcomes  Length of hospital (medical records)  Quality of life (EORTC QLQ-C30 and EORTC QLQ-ELD14)  Destination of discharge from the hospital (NR)  Patient-reported symptoms (The Postoperative Recovery Profile)  Delirium (Confusion Assessment Method)  Lower extremity strength (30-second chair stand)  Maximal inspiratory muscle strength (MicroRPM)  Level of independence in daily living (The ADL-staircase)  Physical activity level (Physical Activity Scale for the Elderly)  Mortality (medical records) |
| **Starting date** | Start date: May 2021  Completion date: June 2025 |
| **Contact information** | Correspondence: Elisabeth Rydwik, Assoc prof  Tel: +4652488818  E-mail: [elisabeth.rydwik@ki.se](mailto:elisabeth.rydwik@ki.se)  Karolinska Institutet Locations, Huddinge, 14183, Sweden |
| **Notes** | Status: Recruiting  The trial was registered prospectively at ClinicalTrials.gov (NCT04878185) on May 7, 2021  <https://clinicaltrials.gov/ct2/show/NCT04878185> |

# **Sebio 2022**

| **Trial name or title** | Prehabilitation Program Based on Health Education and Nordic Walking to Reduce Musculoskeletal Impairments in Women Undergoing Breast Cancer Surgery |
| --- | --- |
| **Objective** | To assess the efficacy of a prehabilitation program consisting of health education and a supervised nordic walking-based intervention in women diagnosed with breast cancer currently undergoing neoadjuvant chemotherapy to decrease post-operative musculoskeletal impairments and improve functionality of the affected arm. |
| **Methods** | Design: parallel Assignment (2 groups) |
| **Participants** | Inclusion:   - Diagnosis of breast cancer scheduled for surgery with or without lymphadenectomy - Candidates to receive neoadjuvant chemotherapy - Not currently undergoing any other physical therapy therapies or treatments   Exclusion:   - Cognitive impairment or inability to read Catalan or Spanish - Prior diagnosis of shoulder impairment or injury - Major musculoskeletal, neurological or cardiorespiratory limitations to prevent participation in the exercise program |
| **Interventions** | Group 1: Nordic walking exercise program group  Group 2: Usual care  Estimated Enrollment: 64 participants in total |
| **Outcomes** | Disabilities of the Arm Shoulder and Hand Questionnaire (Quick DASH), disabilities of the Arm Shoulder and Hand Questionnaire (Quick DASH), disabilities of the Arm Shoulder and Hand Questionnaire (Quick DASH), functionality of the affected upper arm (DASH questionnaire), disabilities of the Arm Shoulder and Hand Questionnaire (Quick DASH), and functionality of the affected upper arm (DASH questionnaire) (Primary outcome)  Secondary outcomes  Upper arm volume (tape measure)  Range of Movement - ROM (goniometer)  Pain severity (Visual Analog Scale)  Health-Related Quality of Life (EORTC QLQ C30).  Handgrip Strength (hydraulic dynamometer Jamar).  Functional capacity (6-Minute Walk Test)  Adherence (as percentage of attended vs. scheduled)  Physical Activity (IPAQ) |
| **Starting date** | Start date: January 2022  Completion date: November 2023 |
| **Contact information** | Correspondence: Anabel Casanovas Álvarez, MSc; Raquel Sebio, PhD  Tel: +34931696573 ext 10340  E-mail: [acasanovasa@tecnocampus.cat](mailto:acasanovasa@tecnocampus.cat); [rsebio@tecnocampus.cat](mailto:rsebio@tecnocampus.cat)  Hospital Santa Creu i Sant Pau, Barcelona, 08041, Spain |
| **Notes** | Status: Recruiting  The trial was registered prospectively at ClinicalTrials.gov (NCT05216302) on January 31, 2022  <https://clinicaltrials.gov/ct2/show/NCT05216302> |

# **Cantarero-Villanueva 2018**

| **Trial name or title** | Attenuating Cancer Treatment-related Toxicity in Oncology Patients with a Tailored Physical Exercise Program: Study Protocol of the ATOPE Trial in Women Recently Diagnosed With Breast Cancer |
| --- | --- |
| **Objective** | To determine if therapeutic exercise before anticancer treatment will mitigate the onset or extent of cardiotoxicity comparing to therapeutic exercise performed during anticancer treatment. |
| **Methods** | Design: parallel Assignment (2 groups) |
| **Participants** | Inclusion:   - 18 years or older - Breast cancer diagnosis I-III stage - On the waiting list to anticancer medical treatment (at least surgery, chemotherapy, and radiotherapy. - If they meet other criteria which predisposes to higher toxicity. - Have signed informed consent. - Have medical clearance for participation.   Exclusion:   - Patient underwent previous cancer treatments. - Patients were previously diagnosed with cancer. - Pregnant patients. - Patients performing other type of therapeutic exercise at diagnosis time with an intake >or = to 150 moderate-intensity or 75 min of vigorous-intensity a day. - Therapeutic exercise practice not recommended because psychiatric or cognitive disorders or cute or chronic condition that prevents exercise (advanced lung disease, oxygen requirement, stenosis >70%, metastasis etc.). |
| **Interventions** | Group 1: Therapeutic exercise before medical treatment group  Group 2: Therapeutic exercise during medical treatment  Estimated Enrollment: 110 participants in total |
| **Outcomes** | Left ventricular ejection fraction (echocardiography) (Primary outcome)  Secondary outcomes  Cardiovascular events (Number of cardiovascular events registered in a diary)  Resting heart rate (Holter)  Heart Rate variability (Holter)  Muscle loss (Inbody)  Quality of life with the Quality of Life (EORTC QLQ-C30)  Quality of life with the Quality of life (QLQ- BR23)  Chemotherapy regimen (type of chemotherapy agent used and number of sessions)  Chemotherapy doses modifications (delay of doses or reduction, total doses received from the total expected)  Early session termination (Patient stopped receiving chemotherapy before last >=1 sessions)  Adverse effects of chemotherapy (>=3 missing consecutive bouts of exercise)  Number of hospitalizations (>=1 bout that required a dose modification during the program and number of bouts modified in total)  Time to treatment failure (NR)  Program adverse effects reported by the participants (Frequency of serious and non-serious)  Functional capacity (6-Minute Walking Test)  Handgrip strength (digital dynamometer)  Shoulder strength (isokinetic dynamometer)  Lower limb strength (isokinetic dynamometer)  Abdominal strength (isokinetic dynamometer)  Flexibility (Modified sit-and-reach test)  Waist and hip circumferences (inelastic tape)  Body composition (InBody)  Oxidative stress (Thiobarbituric acid reactive substances)  Oxidative stress (carbonyls and 8-hydroxy-2' -deoxyguanosine)  Inmune function (cluster of differentiation (CD) 8 and 4 and regulatory T lymphocytes)  inflammation (C-reactive protein (CRP), interleukin (IL) 6 and 10, tumor necrosis factor (TNF) alpha, insulin-like growth factor 1 (IGF-1) |
| **Starting date** | Start date: December 2018  Completion date: December 2022 |
| **Contact information** | Correspondence: Irene Cantarero-Villanueva, PhD  Tel: 958248764  E-mail: [irenecantarero@ugr.es](mailto:irenecantarero@ugr.es)    University of Granada, Granada, 18016, Spain |
| **Notes** | Status: Recruiting  The trial was registered prospectively at ClinicalTrials.gov (NCT03787966) on December 27, 2018  <https://clinicaltrials.gov/ct2/show/NCT03787966>  Publications automatically indexed to this study by ClinicalTrials.gov Identifier (NCT Number):  Postigo-Martin P, Gil-Gutierrez R, Moreno-Gutierrez S, Lopez-Garzon M, Gonzalez-Santos A, Arroyo-Morales M, Cantarero-Villanueva I. mHealth system (ATOPE+) to support exercise prescription in breast cancer survivors: a reliability and validity, cross-sectional observational study (ATOPE study). Sci Rep. 2022 Sep 8;12(1):15217. doi: [10.1038/s41598-022-18706-7](https://pubmed.ncbi.nlm.nih.gov/36076044/).  Postigo-Martin P, Penafiel-Burkhardt R, Gallart-Aragon T, Alcaide-Lucena M, Artacho-Cordon F, Galiano-Castillo N, Fernandez-Lao C, Martin-Martin L, Lozano-Lozano M, Ruiz-Vozmediano J, Moreno-Gutierrez S, Illescas-Montes R, Arroyo-Morales M, Cantarero-Villanueva I. Attenuating Treatment-Related Cardiotoxicity in Women Recently Diagnosed With Breast Cancer via a Tailored Therapeutic Exercise Program: Protocol of the ATOPE Trial. Phys Ther. 2021 Mar 3;101(3):pzab014. doi: [10.1093/ptj/pzab014](https://pubmed.ncbi.nlm.nih.gov/33528004/). |

***References to ongoing studies***

# **Dalton 2020**

Dalton S. Pre-operative Exercise During Neoadjuvant Chemotherapy in Patients With Breast Cancer (Neo-Train). ClinicalTrials.gov 2020 (Recruitment status: Recruiting). [NCT04623554]

# **Palma 2020**

Palma S. Prehabilitation in Patients Suffering From Gynecological Cancers. ClinicalTrials.gov 2020 (Recruitment status: Unknown). [NCT04618094]

# **Fernandez-Bobadilla 2020**

Fernandez-Bobadilla JC. The SEHNeCa Supervised Exercise Project (SEHNeCa). ClinicalTrials.gov 2020 (Recruitment status: Active, not recruiting). [NCT04658706]

# **Behrens 2020**

Behrens J. Resistance training as prehabilitation exercise in Mamma-CA. ICTRP 2020 (Recruitment status: Recruiting). [DRKS00023243]

# **Pameijer 2021**

Pameijer CR. Exercise Intervention Prior to CRS-HIPEC: Feasibility & Impact. ClinicalTrials.gov 2021 (Recruitment status: Recruiting). [NCT04731441]

# **Lin 2021**

Lin CC. A Prehabilitation Program to Boost Postoperative Functional Capacity in Surgical Lung Cancer Patients. ClinicalTrials.gov 2021 (Recruitment status: Recruiting). [NCT04826835]

# **Rydwik 2021**

Rydwik E. Optimizing Physical Function Before Cancer Surgery in Older People at Risk (CanOptiPhys). ClinicalTrials.gov 2021 (Recruitment status: Recruiting). [NCT04878185].

# **Sebio 2022**

Sebio R. Prehabilitation for Breast Cancer Surgery. ClinicalTrials.gov 2022 (Recruitment status: Recruiting). [NCT05216302]

# **Cantarero-Villanueva 2018**

*Cantarero-Villanueva I. Attenuating Cancer Treatment-related Toxicity in Oncology Patients With a Tailored Physical Exercise Program: Study Protocol of the ATOPE Trial in Women Recently Diagnosed With Breast Cancer. ClinicalTrials.gov 2018 (Recruitment status: Recruiting). [NCT03787966]

Postigo-Martin P, Peñafiel-Burkhardt R, Gallart-Aragón T, et al. Attenuating Treatment-Related Cardiotoxicity in Women Recently Diagnosed With Breast Cancer via a Tailored Therapeutic Exercise Program: Protocol of the ATOPE Trial. Phys Ther. 2021;101(3):pzab014. doi:[10.1093/ptj/pzab014](https://doi.org/10.1093/ptj/pzab014)

Postigo-Martin P, Gil-Gutiérrez R, Moreno-Gutiérrez S, et al. mHealth system (ATOPE+) to support exercise prescription in breast cancer survivors: a reliability and validity, cross-sectional observational study (ATOPE study). Sci Rep. 2022;12(1):15217. Published 2022 Sep 8. doi:[10.1038/s41598-022-18706-7](https://doi.org/10.1038/s41598-022-18706-7)

A proof-of-concept evaluation of a perioperative, modular and audiovisual pelvic floor training for patients with a histologically confirmed prostate carcinoma as part of a robot-assisted, laparoscopic or open surgical radical prostatectomy.
