# Supplementary material for: Prehabilitation programs for individuals with cancer: a systematic review of randomized-controlled trials
Source: Syst Rev. 2023 Nov 17;12:219. doi: 10.1186/s13643-023-02373-4 (PMC10655304; doi:10.1186/s13643-023-02373-4)
Supplement: Supplementary file 5 — Additional file 5. Characteristics of the prehabilitation programs (n = 25). [file 13643_2023_2373_MOESM5_ESM.docx]

**Additional file 5**. Characteristics of the prehabilitation programs (n = 25)

| **Study ID** | **Type training** | **Duration (minutes)**  **Length (weeks)**  **Frequency (sessions/week)** | **Components** | **Sessions before surgery/after surgery** | **Adherence**  **(%)** | **Intensity** | **Setting** | **Supervised** | **Multidisciplinary team** |
| --- | --- | --- | --- | --- | --- | --- | --- | --- | --- |
| Banerjee 2018 (47) | Int: aerobic training (HIIIT)  Con: usual care | 40 min  4 weeks  2 session/ week. | 5-10 min warm up         30 min aerobic training (HIIT)  Cool down: NR | 8/NR | NR | Aerobic training (HIIT): 70–85% predicted MHR on 220-age) | Clinic/  Hospital | Supervised | Small team of exercise science staff working closely with the study urologist |
| Berkel 2020 (29) | Int: combined training (HIIT and resistance training)  Con: usual care | 60 min  3 weeks.  3 session/ week. | Aerobic training 40 min (7min warm up, 30min HIIT, 3min cool down  First week: 120 seg of work and 180 recovery.  Second week: 140 seg of work and 160 recovery.  Third week: 160 seg of work and 140 recovery.  Resistance training: 3 sets, 8 reps | 9 | 90% | Aerobic training (HIIT) :120% of the work rate and active recovery at 50%)  Resistance training: 70%- 82% 1RM | Mixed (home/clinic) | Partially | Therapists/ nurse |
| Bousquet-Dion 2018 (30) | Int: combined training (MICT and resistance training)  Con: rehabilitation | 65 min  4 weeks  3-4 session/ week | 5 min warm-up    30 min aerobic training   25 min resistance training   5 min stretching | 12-16 / 8 | 98% | Aerobic training:(MICT) 60 - 70% MHR   Resistance training was progressed   to mild exertion (defined as 12 or less on the 20-point Borg scale) | Mixed (home/clinic) | Partially | Kinesiologist,   nutritionist and a psychology-trained research team member |
| Carli 2010 (32) | Int: combined training (MICT and resistance training)  Con: rehabilitation (walk/breathing group) | 45 min  4 weeks  3 session/ week. | 20-30 min aerobic training  10-15 min resistance training | 12 / NR | 16% | Aerobic training (MICT): 50% MHR  Resistance training: 8 -12 RM | Home | Partially | Medical research team |
| Carli 2020 (31) | Int: combined training (MICT and resistance training)  Con: rehabilitation | 65 min  4 weeks  4 session/ week | 5 min warm-up    30 min aerobic training   25 min resistance training   5 min stretching | 16 / 16 | 68% | Aerobic training (MICT): Moderate intensity  Resistance training: NR | Mixed (home/clinic) | Partially | Kinesiologist,   nutritionist and a psychology-trained research team member |
| Centemero 2010 (44) | Int: pelvic floor training  Con: rehabilitation with pelvic floor training | 60 min  8 weeks  2 session/ week | NR | 8 / 8 | NR | NR | Clinic/Hospital | Partially | Therapist and surgeon |
| Dronkers, 2010 (13) | Int: combined training (MICT and resistance training) plus respiratory muscle training.  Con: rehabilitation (home-based exercise advice plus inspiratory muscle training advice) | 45min  2-4 weeks  2 session/ week | warm-up resistance training of the lower limb extensors.  15 minutes inspiratory muscle training  20-30 min aerobic training  training funtional | 5 / NR | 97% | Aerobic training (MICT): 55–75% MHR  Resistance training: 60–80% RM (8–15 RM)  Respiratory muscule training 10–60% MIP | Mixed (home/clinic) | Supervised | Therapists |
| Dunne 2016 (33) | Int: aerobic training (HIIT)  Con: usual care | 30 min  4 weeks  3 session/ week | 30 min interval training alternating between moderate and vigorous intensity | 12 / NR | 90% | Aerobic training (HIIT) 60%-70% MHR | Clinic/Hospital | Supervised | Medical research team |
| Gillis 2014 (34) | Int: combined training (MICT and resistance training)  Con: rehabilitation | 50 min  4 weeks  3 session/ week | 5 min warm-up  20 min aerobic training  20 min resistance training  5 min cool-down | 12 / 24 | 78% | Aerobic training:(MICT) 40% HRR  Resistance training: 8-12 RM | Home | Unsupervised | Kinesiologist,   nutritionist and a psychology-trained research team member |
| Gloor, 2022 (35) | Int: combined training (HIIT and resistance training)  Con: usual care | 90 min  3-6 weeks  3 session/ week | Warm-up Aerobic (training, HIIT), Resistance training Cool-down: stretching. | 3 to 6/NR | NR | Aerobic training (HIIT):85-90% of maximum training capacity  Resistance training: NR | Mixed (home/clinic) | Supervised | Therapists |
| Heiman 2021 (49) | Int: aerobic training (MICT)  Con: usual care | 30 min  2 week  2 week | NR | 14/28 | NR | Aerobic training (MICT): Medium | Unclear | Unsupervised | Therapists/ nurse |
| Karlsson 2019 (36) | Int: combined training (HIIT and resistance training) plus inspiratory muscle training  Con: usual care | 60 min  2 week  2–3 session/ week | Inspiratory muscle training, aerobic training HIIT, resistance training (chair stands and step-up with weight belts 3x10repetitions) | 6 - 8/NR | 97% | Aerobic training (HIIT): 5–8 /10 Borg  Resistance training: 7/10 Borg  Inspiratory muscle training (50% of maximal capacity) | Home | Supervised | Therapists |
| Lai 2017 (42) | Int: aerobic training (MICT) plus respiratory muscles training  Con: usual care | 60 min  1 weeks  3 session/ week | 30 min respiratory exercises  30 min aerobic training | 7 / 0 | 53% | NR | Clinic/  Hospital | Supervised | Therapists |
| Laurienzo 2013 (28) | Int: pelvic floor training  Con 1: pelvic floor training plus electrical stimulation  Con 2: usual care | NR | NR | 10 / NR | NR | NR | NR | NR | Therapist and surgeon |
| Licker 2016 (39) | Int: combined training (HIIT and resistance training)  Con: usual care | 30 min  NR  2-3 session /week | 5 min warm-up  two 10 min series of 15 sec sprint intervals interspersed by 15 sec pauses and a 4 min rest between the two series.  5 min active recovery period | NR / NR | 87% | Aerobic training (HIIT): 80%–100% peak WR  Resistance training: NR | Clinic/  Hospital | Supervised | Therapists |
| Liu 2019 (40) | Int: combined training (MICT and resistance training)  Con: usual care | 40 min    2 weeks  3 session / week | 5 min warm-up  25 min aerobic training  10 min resistance training  with an interval of 2 min between  each set | 6 / NR | NR | Aerobic training (MICT): 60%-70% MHR  Resistance: Borg scale score of 13-16 | Home | Unsupervised | Therapist, surgeon, and psychiatrist |
| Moug 2019 (37) | Int: aerobic training (MICT)  Con: usual care | 30 min  14 week  5 session /week | 30 minutes aerobic training | 70 / NR | 75% | NR | Home | Partially | NR |
| Ocampo-Trujillo 2014 (46) | Int: pelvic floor training  Con: usual care | NR | NR | 12 / 0 | NR | NR | Clinic/  Hospital | Supervised | NR |
| Onerup 2022 (38) | Int: aerobic training (MICT) plus inspiratory muscle training (IMT)  Con: usual care | 30 min  2 week  7 session / week | Aerobic training (MICT) 30 min.  Respiratory muscle training: 30 x 2 breaths. | 14 / 28 | NR | Aerobic training (MICT):  12-14/ 20 Borg.  Inspiratory muscle training: 30% of maximal inspiratory pressure | Home | Unsupervised | Therapists/ nurse |
| Pehlivan 2011  (33) | Int: aerobic training (MICT) plus respiratory muscles training  Con: usual care | NR | NR | 2 / NR | NR | NR | Clinic/  Hospital | Supervised | NR |
| Peng 2021 (50) | Int: resistance training plus respiratory muscle training  Con: usual care | NR | NR | NR | 92%-100% | NR | Mixed (home/clinic) | Partially | Therapists |
| Santa Mina 2018 (45) | Int: combined training (MICT and resistance training)  Con: pelvic floor training | 60 min  5 weeks  3-4 session/ week | 5 min warm-up  25 min aerobic training  25 min resistance training  5 min cool-down | NR / NR | 68% | Aerobic training (MICT): 40-60% HRR /  Resistance training: 8-12 RM | Home | unsupervised | NR |
| Sebio-García 2017 (43) | Int: combined training (MICT and resistance training)  Con: usual care | 30 min  NR  3-5 session/ week | 5 min warm-up  21 min aerobic training  4 min cool-down | 16 (range 8–25) / NR | 50% | Aerobic training (MICT): 30-80 W peak Resistance training: NR | Mixed (home/clinic) | Supervised | Therapists and researchers |
| Steffens 2021 (48) | Int: combined training (MICT and resistance training).  Con: usual care. | 60 min               2-6 week  1-4 session/ week | 10 min warm-up  40 min (aerobic training, respiratory, resistance training),  10 cool-down. | 2-24 /NR | 93% supervised, 64% no supervised | Aerobic training (MICT): 12 to 14 Borg  Resistance training 40 to .60% 1 RM | Mixed (home/clinic) | Partially | Therapists |
| Yamana 2015 (51) | Combined training (MICT and Resistance) plus respiratory rehabilitation muscle. | 60 min  NR  7 session/ week | 20 min Combined training (MICT: biking on an ergometer.  Resistance training lower limbs and abdominal muscle.  respiratory rehabilitation muscle. | NR | NR | NR | Clinic/  Hospital | Supervised | Therapists |
|  |  |  |  |  |  |  |  |  |  |

 High-intensity interval training, HIIT; Maximum heart rate, MHR; Not reported; NR; Heart rate reserve, HRR, maximal inspiratory pressure, MIP; Minutes: min; Moderate intensity continuous training, MICT; Repetition maximum, RM; Work rate, WR.
