# Supplementary material for: Prehabilitation programs for individuals with cancer: a systematic review of randomized-controlled trials
Source: Syst Rev. 2023 Nov 17;12:219. doi: 10.1186/s13643-023-02373-4 (PMC10655304; doi:10.1186/s13643-023-02373-4)

**Additional file 6. Subgroup analysis for the completeness of reporting of the exercise training interventions in the prehabilitation programs**

***Completeness of reporting by Exercise modality***

Combined training was the most studied exercise intervention (14, 56%). Furthermore, half of the total items score was ≥72% of completeness report.

The interventions with aerobic exercise were categorized as moderate intensity aerobic training (MICT) which represented the second most reported exercise modality with at least 12 items that registered a score of <60%, whereas high intensity Interval training (HIIT) was the least studied (2, 8%) of the interventions with aerobic exercise and had a 100% completeness report in the items 1, 4, 5, 11, 12, 13, 14a, 14b and 16b.

The completeness of report in the interventions with pelvic floor training (3, 12%) only had a score of 67% in items 2, 4 and 13. In contrast, with the exception of items 7b, 8 and 10, resistance training modality obtained 100% in the completeness of the report.

***Subgroup analysis 1: colorectal cancer***

Overall, the reporting of interventions in the 12 studies for colorectal cancer showed a score ≥84 in the items 1, 4, 5, 12, 13, 14b, 16a.

The remaining items ranged between 9% and 75%.

***Subgroup analysis 2: lung cancer***

In exercise interventions for lung cancer, items 1, 2, 13 range between 80% 100%, while almost half of the items (5, 6, 7a, 7b, 8, 11, 14b, 15, 16a) had a score with a range between 0% and 40%.

***Subgroup analysis 3: prostate cancer***

Almost half of the items (2, 3, 8, 9,10, 11, 15, 16a, 16b) in the interventions for prostate cancer had a score with a range between 0% and 50% and only one items 4 and 13 obtained a score ≥72

.

| **CERT item** | **Number of interventions that completed the report n=25 (%)** | **Type of cancer** | | | | | | |
| --- | --- | --- | --- | --- | --- | --- | --- | --- |
|  |  | **Colorectal cancer (n=12)** | **Lung cancer (n=5)** | **Prostate cancer (n=4)** | **Breast cancer (n=1)** | **Bladder**  **Cancer (n=1)** | **Esophageal cancer (n=1)** | **Abdominal cancer (n=1)** |
| Item 1. What (materials) | 20 (80%) | 11 (92%) | 4 (80%) | 2 (50%) | 0 (0%) | 1 (100%) | 1 (100%) | 1 (100%) |
| Item 2. Who (provider) | 17 (68%) | 8 (67%) | 4 (80%) | 2 (50%) | 1 (100%) | 1 (100%) | 0 (0%) | 1 (100%) |
| Item 3. Individually or in a group | 14 (56%) | 9 (75%) | 2 (40%) | 1 (25%) | 1 (100%) | 0 (0%) | 0 (0%) | 1 (100%) |
| Item 4. Supervised or unsupervised | 21 (84%) | 12 (100%) | 3 (60%) | 3 (75%) | 0 (0%) | 1 (100%) | 1 (100%) | 1 (100%) |
| Item 5. Adherence report | 17 (68%) | 11 (92%) | 1 (20%) | 2 (50%) | 1 (100%) | 1 (100%) | 0 (0%) | 1 (100%) |
| Item 6. Motivation strategies | 11 (44%) | 7 (59%) | 0 (0%) | 2 (50%) | 1 (100%) | 0 (0%) | 0 (0%) | 1 (100%) |
| Item 7a. Exercise progression | 14 (56%) | 8 (67%) | 2 (40%) | 2 (50%) | 0 (0%) | 1 (100%) | 0 (0%) | 1 (100%) |
| Item 7b. Program progression | 11 (44%) | 7 (59%) | 1 (20%) | 2 (50%) | 0 (0%) | 1 (100%) | 0 (0%) | 0 (0%) |
| Item 8. Exercise replication | 2 (8%) | 1 (9%) | 0 (0%) | 0 (0%) | 0 (0%) | 1 (100%) | 0 (0%) | 0 (0%) |
| Item 9. Home components | 12 (48%) | 8 (67%) | 2 (40%) | 1 (25%) | 0 (0%) | 0 (0%) | 0 (0%) | 1 (100%) |
| Item 10. Non exercise components | 8 (32%) | 5 (42%) | 2 (40%) | 1 (25%) | 0 (0%) | 0 (0%) | 0 (0%) | 0 (0%) |
| Item 11. Adverse events report | 12 (48%) | 7 (59%) | 1 (20%) | 1 (25%) | 0 (0%) | 1 (100%) | 1 (100%) | 1 (100%) |
| Item 12. Setting | 19 (76%) | 11 (92%) | 3 (60%) | 2 (50%) | 0 (0%) | 1 (100%) | 1 (100%) | 1 (100%) |
| Item 13. Description of the exercise | 24 (96%) | 12 (100%) | 5 (100%) | 3 (75%) | 1 (100%) | 1 (100%) | 1 (100%) | 1 (100%) |
| Item 14a. Exercises generic or tailored? | 14 (56%) | 7 (59%) | 3 (60%) | 2 (50%) | 0 (0%) | 1 (100%) | 0 (0%) | 1 (100%) |
| Item 14b. Description of the adaptation made in the exercises | 16 (64%) | 10 (84%) | 2 (40%) | 1 (25%) | 0 (0%) | 1 (100%) | 1 (100%) | 1 (100%) |
| Item 15. Rules for starting level | 6 (24%) | 5 (42%) | 0 (0%) | 0 (0%) | 0 (0%) | 0 (0%) | 0 (0%) | 1 (100%) |
| Item 16a. How adherence to exercise was measured | 14 (56%) | 10 (84%) | 2 (40%) | 1 (25%) | 0 (0%) | 0 (0%) | 0 (0%) | 1 (100%) |
| Item 16b. Is the intervention carried out according to how it was planned? | 15 (60%) | 9 (75%) | 3 (60%) | 1 (25%) | 0 (0%) | 1 (100%) | 0 (0%) | 1 (100%) |

| **CERT item** | **Number of interventions that completed the report n=25 (%)** | **Exercise modality** | | | | |
| --- | --- | --- | --- | --- | --- | --- |
|  |  | **Combined training**  **(n= 14)** | **Aerobic training**  **MICT**  **(n= 5)** | **Aerobic training**  **HIIT**  **(n= 2)** | **Pelvic floor training (n= 3)** | **Resistance training**  **(n= 1)** |
| Item 1. What (materials) | 20 (80%) | 13 (93%) | 3 (60%) | 2 (100%) | 1 (34%) | 0 (0%) |
| Item 2. Who (provider) | 17 (68%) | 11 (79%) | 3 (60%) | 1 (50%) | 2 (67%) | 1 (100%) |
| Item 3. Individually or in a group | 14 (56%) | 10 (72%) | 3 (60%) | 1 (50%) | 0 (0%) | 0 (0%) |
| Item 4. Supervised or unsupervised | 21 (84%) | 13 (93%) | 3 (60%) | 2 (100%) | 2 (67%) | 1 (100%) |
| Item 5. Adherence report | 17 (68%) | 10 (72%) | 3 (60%) | 2 (100%) | 1 (34%) | 1 (100%) |
| Item 6. Motivation strategies | 11 (44%) | 7 (50%) | 2 (40%) | 0 (0%) | 1 (34%) | 1 (100%) |
| Item 7a. Exercise progression | 14 (56%) | 10 (72%) | 2 (40%) | 1 (50%) | 1 (34%) | 0 (0%) |
| Item 7b. Program progression | 11 (44%) | 8 (57%) | 1 (20%) | 1 (50%) | 1 (34%) | 0 (0%) |
| Item 8. Exercise replication | 2 (8%) | 0 (0%) | 0 (0%) | 1 (50%) | 0 (0%) | 1 (100%) |
| Item 9. Home components | 12 (48%) | 10 (72%) | 1 (20%) | 0 (0%) | 0 (0%) | 1 (100%) |
| Item 10. Non exercise components | 8 (32%) | 7 (50%) | 0 (0%) | 0 (0%) | 1 (34%) | 0 (0%) |
| Item 11. Adverse events report | 12 (48%) | 7 (50%) | 2 (40%) | 2 (100%) | 0 (0%) | 1 (100%) |
| Item 12. Setting | 19 (76%) | 13 (93%) | 2 (40%) | 2 (100%) | 1 (34%) | 1 (100%) |
| Item 13. Description of the exercise | 24 (96%) | 14 (100%) | 5 (100%) | 2 (100%) | 2 (67%) | 1 (100%) |
| Item 14a. Exercises generic or tailored? | 14 (56%) | 9 (65%) | 1 (20%) | 2 (100%) | 1 (34%) | 1 (100%) |
| Item 14b. Description of the adaptation made in the exercises | 16 (64%) | 11 (79%) | 2 (40%) | 2 (100%) | 0 (0%) | 1 (100%) |
| Item 15. Rules for starting level | 6 (24%) | 5 (36%) | 1 (20%) | 0 (0%) | 0 (0%) | 0 (0%) |
| Item 16a. How adherence to exercise was measured | 14 (56%) | 11 (79%) | 2 (40%) | 0 (0%) | 0 (0%) | 1 (100%) |
| Item 16b. Is the intervention carried out according to how it was planned? | 15 (60%) | 8 (57%) | 3 (60%) | 2 (100%) | 1 (34%) | 1 (100%) |


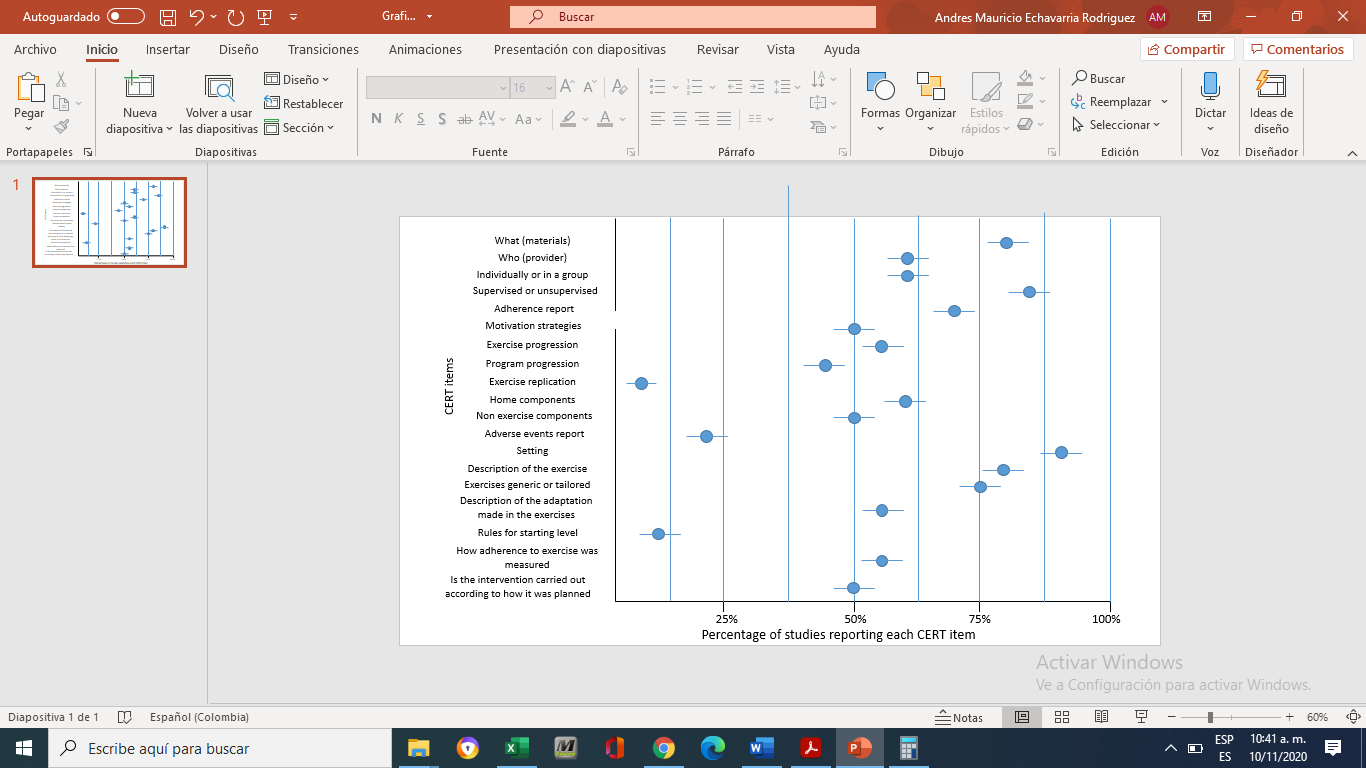

Supplement: Supplementary file 6 — Additional file 6. Subgroup analysis for the completeness of reporting of the exercise training interventions in the prehabilitation programs. [file 13643_2023_2373_MOESM6_ESM.docx]
