# Supplementary material for: Prehabilitation programs for individuals with cancer: a systematic review of randomized-controlled trials
Source: Syst Rev. 2023 Nov 17;12:219. doi: 10.1186/s13643-023-02373-4 (PMC10655304; doi:10.1186/s13643-023-02373-4)
Supplement: Supplementary file 7 — Additional file 7. Other outcome measures reported in the included studies. [file 13643_2023_2373_MOESM7_ESM.docx]

**Additional file 7. Other outcome measures reported in the included studies**

*Mental health outcomes*

- Psychological well-being: six studies (35–39,41)
- Depression and anxiety: one study (48)
- Psychosocial well-being: one study (41)

*Physical health outcomes*

- Aerobic capacity: ten studies (29–31,33,34,35,36,37,39,41)
- Physical functioning: six studies (13, 34,35,38,39,41)
- Energy expenditure: five studies (13, 30,36–38)
- Dyspnea: one study (30)
- Fatigue: three studies (13,30,35)
- Pain: one study (35)
- Gastrointestinal function: one study (50)
- Maximal inspiratory pressure: one study (13)

*Hospital-related outcome measures*

- Hospital expenditures: three studies (29,30,37)
- Hospital readmissions: five studies (29,35,36,37,49)
- Emergency department visits: two studies (36,37)

*Other outcome measures*

- Urinary incontinence: four studies (28,32,40,41)
- Pulmonary function: two studies (33,34)
- Mortality: three studies (35,40,50)
- Patient-reported recovery: one study (36)
